# Shedding New Lights Into STED Microscopy: Emerging Nanoprobes for Imaging

**DOI:** 10.3389/fchem.2021.641330

**Published:** 2021-04-20

**Authors:** Yanfeng Liu, Zheng Peng, Xiao Peng, Wei Yan, Zhigang Yang, Junle Qu

**Affiliations:** Key Laboratory of Optoelectronic Devices and Systems of Ministry of Education and Guangdong Province, College of Optoelectronic Engineering, Shenzhen University, Shenzhen, China

**Keywords:** STED, nanoprobes, bioimaging, subdiffraction imaging, material science

## Abstract

First reported in 1994, stimulated emission depletion (STED) microscopy has long been regarded as a powerful tool for real-time superresolved bioimaging . However, high STED light power (10^1∼3^ MW/cm^2^) is often required to achieve significant resolution improvement, which inevitably introduces phototoxicity and severe photobleaching, damaging the imaging quality, especially for long-term cases. Recently, the employment of nanoprobes (quantum dots, upconversion nanoparticles, carbon dots, polymer dots, AIE dots, etc.) in STED imaging has brought opportunities to overcoming such long-existing issues. These nanomaterials designed for STED imaging show not only lower STED power requirements but also more efficient photoluminescence (PL) and enhanced photostability than organic molecular probes. Herein, we review the recent progress in the development of nanoprobes for STED imaging, to highlight their potential in improving the long-term imaging quality of STED microscopy and broadening its application scope. We also discuss the pros and cons for specific classes of nanoprobes for STED bioimaging in detail to provide practical references for biological researchers seeking suitable imaging kits, promoting the development of relative research field.

## Introduction

Photoluminescence (PL) microscopy imaging has long been a powerful tool in biological research. However, the resolution of conventional far-field fluorescence microscopy was limited to half of the imaging wavelength (∼200 nm) by Abbe’s optical diffraction-limited theory (Rayleigh, 1896). Such resolution soon became insufficient as the interested events in cytobiological research went smaller in space and faster in timescale. In 1990s, Hell and coworkers put forward and realized the idea of stimulated emission depletion (STED) microscopy (Hell and Wichmann, 1994; Klar and Hell, 1999), which provided the world a state-of-art method to perform imaging beyond the diffraction limit.

**FIGURE 1:**
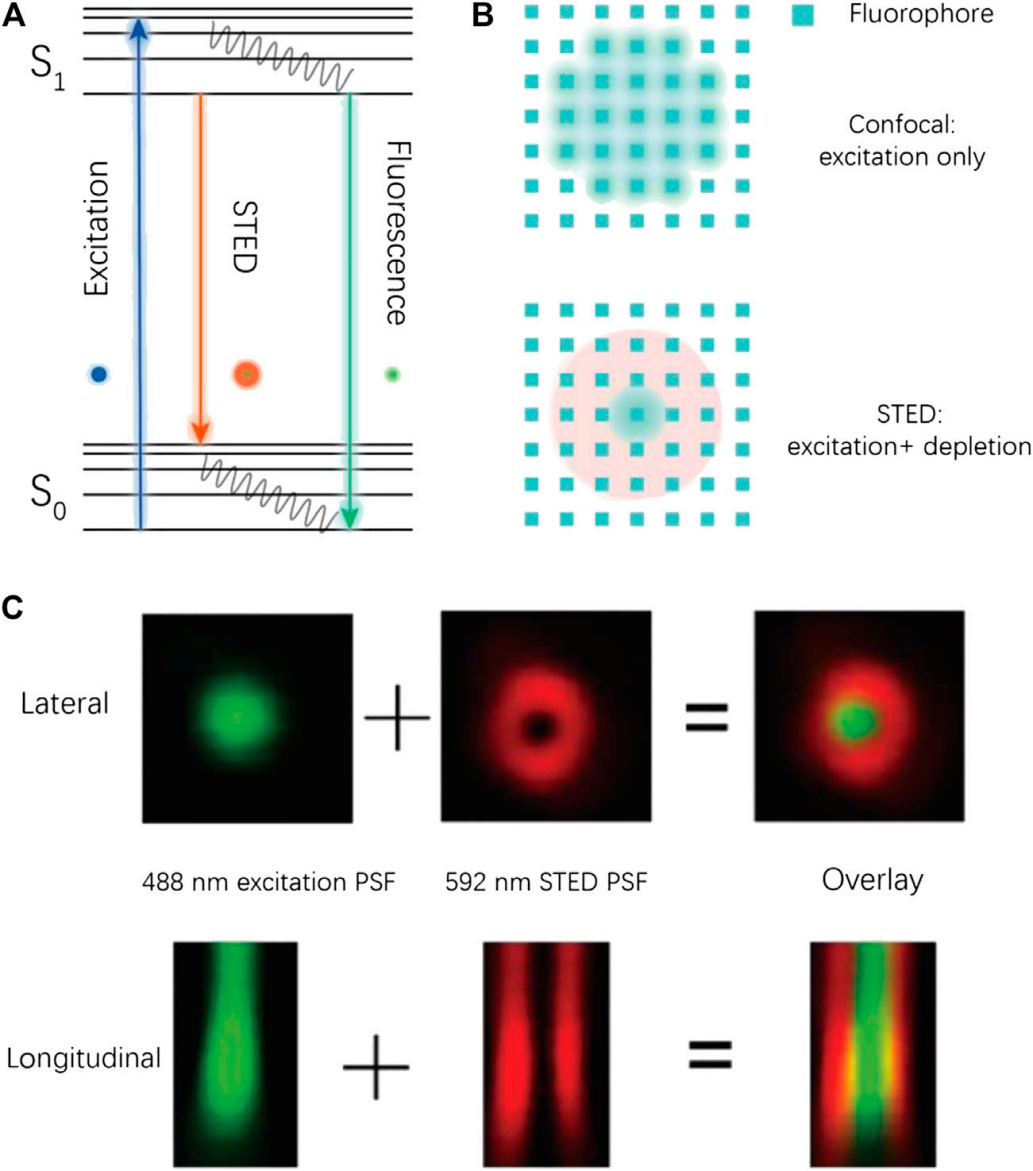
Conceptual illustration for STED imaging. **(A)** The Jablonski diagram for a typical STED process. **(B)** Illustration of the PSF shrinking in STED imaging. **(C)** Experimental PSF in a STED imaging process (Ye et al., 2018).

The system of STED is based on a modification of the preexisting confocal microscopy. In confocal imaging, the size of point spread function (PSF) is limited by optical diffraction (Figure 1). As a result, a number of fluorophores are irradiated at the same time during the acquisition of a single pixel, which leads to a limited resolution. In a STED imaging setup, a donut-like depletion light is applied to suppress the emission of peripheral fluorophores by triggering their stimulated emission (at a red-shifted wavelength), which effectively decreases the size of PSF and thus ensures subdiffraction imaging (see Figure 1).

Compared with other superresolution imaging methods like structure illumination microscopy (SIM) (Gustafsson, 2000; Gustafsson, 2005), photoactivated localization microscopy (PALM) (Betzig et al., 2006), and stochastic optical reconstruction microscopy (STORM) (Rust et al., 2006; Huang et al., 2008), STED is a pure-optical measure that can be applied with a variety of dyes, while being free from complex postimaging calculation. These advantages make STED favorable for superresolution imaging in a real-time mode.

However, the improvement of resolution also comes with a price: depletion of most conventional fluorescent labels, such as molecular probes and fluorescent proteins (FPs), generally requires very high depletion light intensity (Hein et al., 2008; Meyer et al., 2008). As a result, STED bioimaging suffers from severe photobleaching and phototoxicity (Wildanger et al., 2009), thus hampering the development of their long-term imaging applications with live samples. Fortunately, the development of material science has provided a vast menu of photostable fluorescent nanomaterials (Wolfbeis, 2015; Jin et al., 2018). Generally speaking, these nanomaterials are stable and bright and can be more efficiently depleted than molecular dyes, suggesting their potential utility as antibleaching STED probes.

In this article, we first introduce categories of nanoprobes and their brief history in STED applications. Then, we perform a systematic cross-comparison to discuss the pros and cons of different nanoprobes for STED. In addition, we summarize the major challenges for nanoprobes in STED microscopy and propose an outlook on the future development of nanoprobe-based STED imaging.

## Nanoparticles for STED: Categories and Brief History

**FIGURE 2:**
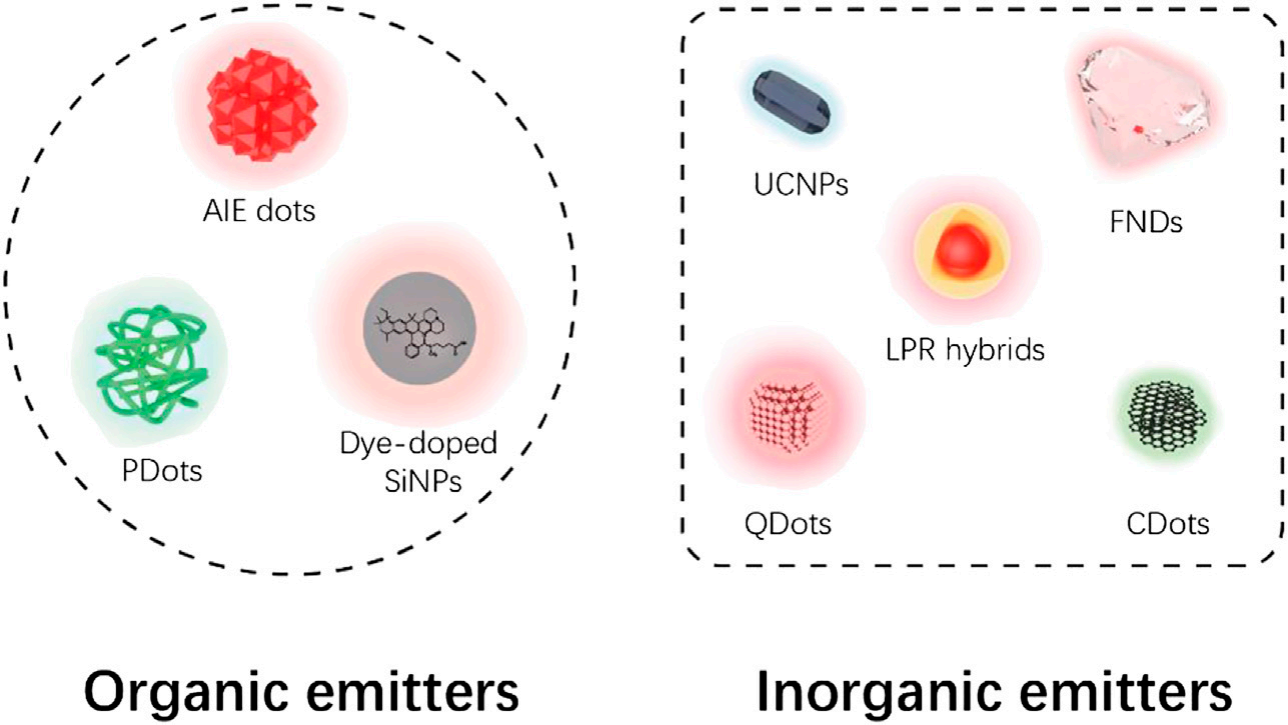
Categories of STED nanoprobes based on current report. Note that dye-doped SiNPs were classified as organic emitters based on their molecular fluorescence origin.

The nanoparticles applied for STED imaging can be divided into two major categories according to their PL origin (Figure 2). Nanoparticles with organic PL origin including aggregation-induced emission (AIE) dots, polymer dots (PDots), and dye-doped silica nanoparticles (SiNPs) have similar emitting mechanism and properties like molecular dyes, but with improved STED performance. Inorganic nanoprobes like fluorescent nanodiamonds (FNDs), localized plasmonic resonance (LPR) hybrids, quantum dots (QDots), upconversion nanoparticles (UCNPs), and carbon dots (CDots) have energy structure and PL properties different from molecular dyes and, in many cases, are more satisfactory emitters with higher depletion efficiencies.

### Organic Emitters

#### Dye-Doped SiNPs

Dye-doped SiNPs are usually classified as organic emitters due to their molecular PL origin, despite their chemical composition with a large amount of inorganic element (Si). For this reason, the basic optical properties of SiNPs such as photostability and depletion efficiency are highly dependent on the character of the doped molecular dyes.

In 2010s, Kraegeloh and coworkers initiated a series of studies on the SiNPs for STED cellular imaging. At the early stage, large SiNPs with diameter exceeding 100 nm were synthesized, which clearly formed discernable aggregates in living cells (Schubbe et al., 2010). Later, SiNPs with smaller particle size were achieved (Schübbe et al., 2012), allowing for a quantitative measurement on their cellular uptake (Peuschel et al., 2015), demonstrating the realistic applicability of these materials. To further synthesize smaller SiNPs with higher brightness, Kraegeloh and coworkers further modified the fabrication technique by applying a dye-conjugated organosilica reagent (Tavernaro et al., 2017). As a result, particles with smaller size (down to 14 nm) and yet higher fluorophore density were fabricated, which showed higher brightness and better photostability for STED imaging with a limiting resolution of 80 nm.

**FIGURE 3:**
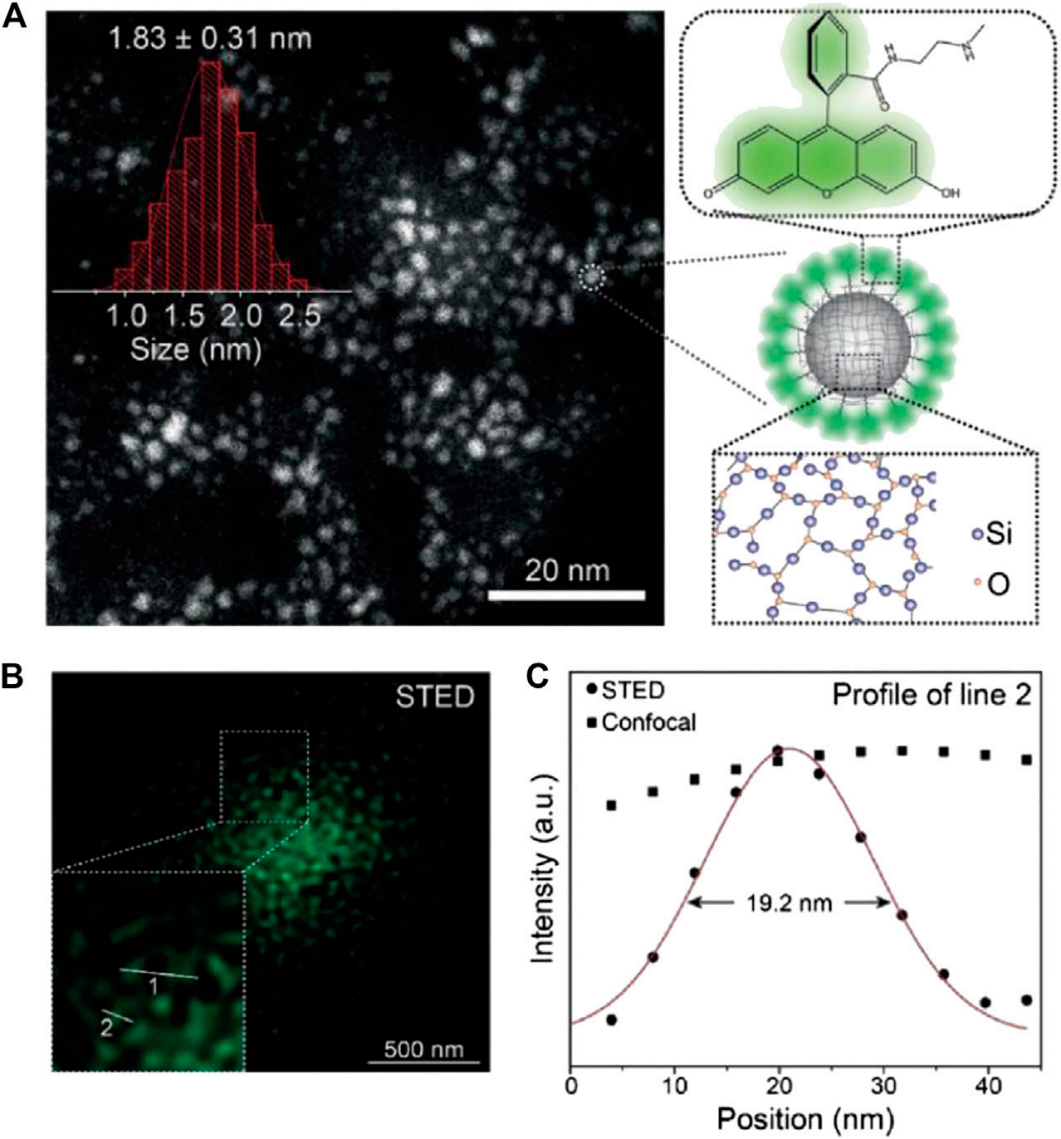
Sub-2-nm dye-doped SiNPs for STED imaging (Liang et al., 2020). **(A)** The morphology and structure illustration of SiNPs. **(B)** The STED microscopy image of dispersed SiNPs. **(C)** The plotfile of signal intensities for confocal and STED images. A resolution of 19.2 nm was calculated from the full width at half maximum (FWHM) of the plotfile.

To achieve higher STED resolution, Qu, Liu, and other coworkers designed and synthesized fluorescent SiNPs by hydrothermally treating saline linkers together with dye molecules (Liang et al., 2020; Figure 3). These as-synthesized sub-2-nm SiNPs showed outstanding photostability and ultrahigh brightness with a photoluminescence quantum yield (PLQY) of 99%, due to the successful elimination of spin–orbit coupling (SOC) of the fluorescein-derived dye. Furthermore, the SiNPs can be effectively depleted with low STED power, which allowed a resolution of up to 19.2 nm (10-fold improved to confocal results) at <40 mW STED power.

Another success in improving resolution and reducing the STED power requirement was achieved by introducing a nonplanar twist intramolecular charge transfer (TICT) compound DAPF as the emitting core (Man et al., 2019). As a result, a stable and highly efficient STED nanoprobe was fabricated, which achieved a lateral resolution of 60 nm at <1 MW/cm^2^ STED intensity (∼3 mW in power scale) for cell imaging.

#### AIE Dots

In 2001, fluorescent molecules with aggregation-induced emission (AIE) feature were first reported by Benzhong Tang and coworkers (Luo et al., 2001). In contrast to conventional aggregation-caused quenching (ACQ) dyes, the AIE fluorophores featured higher emission efficiency in aggregation. Such fascinating character promoted the fast development of the colloidal AIE dots, which have found broad applications in fields of bioimaging, sensing, and theranostics (Hong et al., 2011). According to Tang and coworkers’ pioneer work, the AIE materials are more photostable and showed easier depletion by STED than molecular fluorophores (Yu et al., 2015). To further probe the applicability of AIE dots in STED imaging, Tang et al. designed and synthesized silica-hybridized AIE dots from an AIE molecule TTF (Li et al., 2017). The resultant 24-nm particles reached subdiffraction resolution (∼30 nm) when depleted with a 775-nm STED light (300 mW) and showed a highly stable and nontoxic effect for cellular imaging.

**FIGURE 4:**
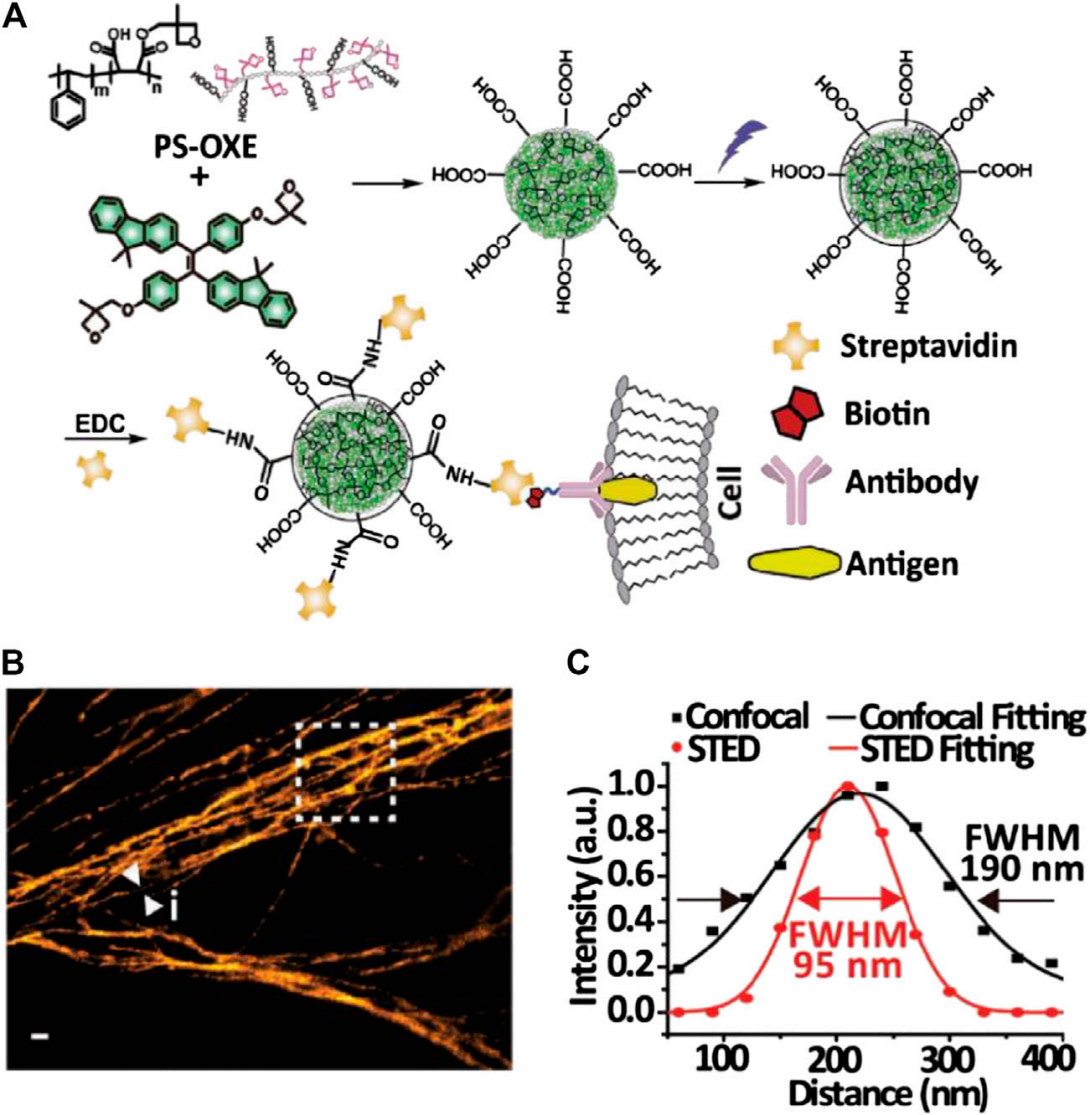
Bioconjugated AIE dots for STED imaging (Fang et al., 2017). **(A)** Synthesis and modification of AIE dots for specific labeling. **(B)** STED microscopy image of microtubules in MCF-7 cells labeled with AIE dots. **(C)** Plotfile for confocal and STED imaging results in the selected area.

The potential of AIE dots for subcellular tracking in both fixed and living cells and *in vivo* imaging was further reported (Li et al., 2018; Fang et al., 2017; Xu et al., 2020). Fang and coworkers synthesized carboxylated AIE dots by nanoprecipitation and conjugated them with streptavidin for the STED imaging of microtubules in fixed MCF-7 cells (Fang et al., 2017; Figure 4). Li et al. synthesized positively charged AIE dots by direct precipitation of an amphiphilic AIE molecule and used it to label the mitochondria in living cells for STED imaging (Li et al., 2018). Xu et al. synthesized highly biocompatible AIE dots with PEG passivation for *in vitro* and *in vivo* STED bioimaging of living cells and fish-tail microvessels (Xu et al., 2020). The imaging resolution in these works reached 70∼100 nm, but the STED power requirement (100∼150 mW) was close to that for small molecular probes.

#### PDots

**FIGURE 5:**
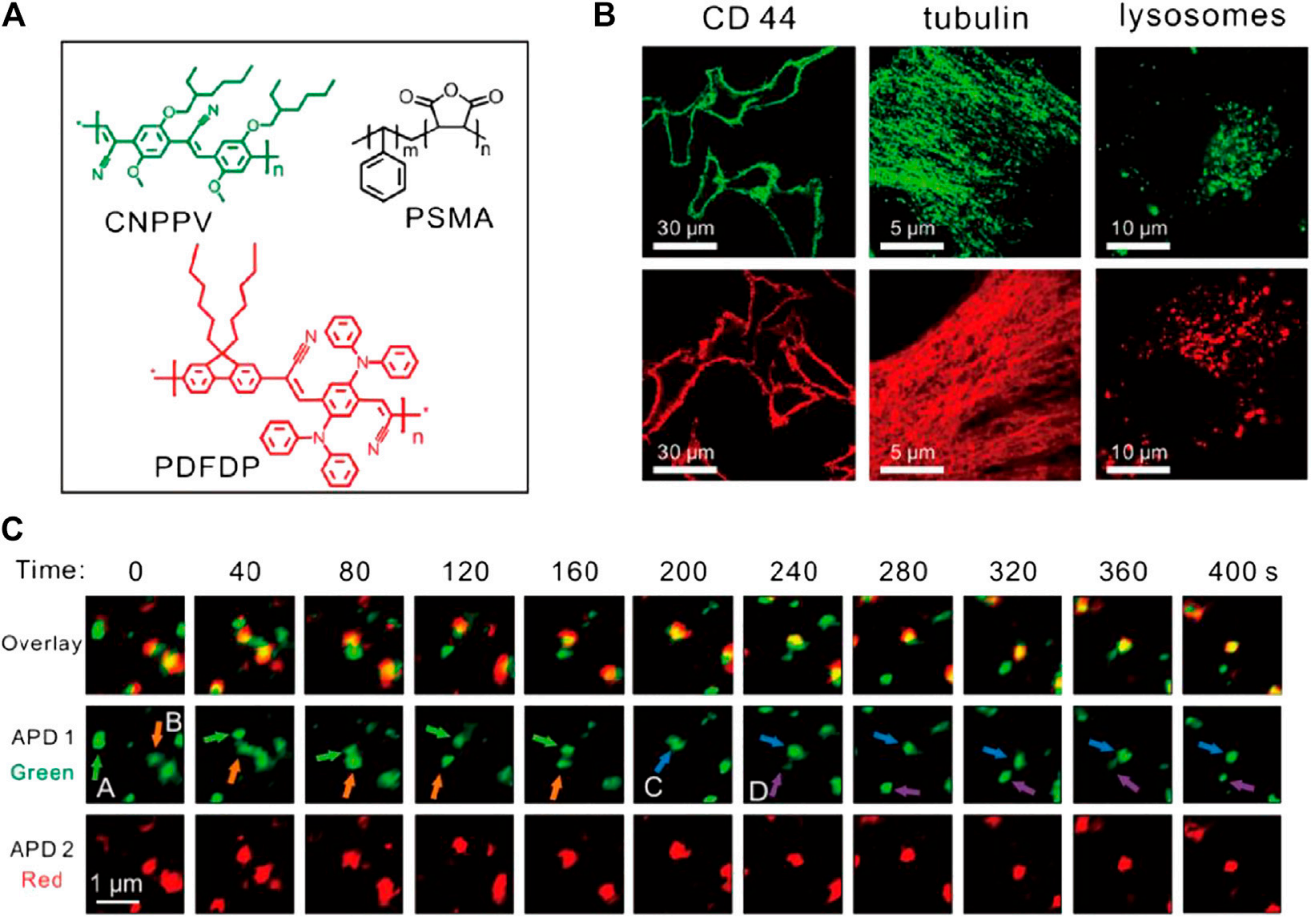
Bioconjugated PDots for STED imaging (Wu et al., 2020). **(A)** Chemical composition of two PDots. **(B)** STED imaging of subcellular structures bonded with PDots through immunofluorescence labeling. **(C)** Application of PDots for real-time tracking of organelle interactions.

PDots, an emerging class of nanoparticles derived from fluorescent semiconductor polymers like poly(9, 9-dioctylfluorene-co-benzothiadiazole) (PFBT) and poly(1,4-phenylenevinylene) (PPV), are ideal bioimaging probes with high brightness, photobleaching resistance, and low toxicity (Wu et al., 2010). Despite the scarcity of relative reports, PDots have proven high potential for STED imaging applications. In 2018, Wu and Fang et al. first adopted 40 nm-sized PDots for STED imaging (Wu Y. et al., 2018). As expected, these particles were highly biocompatible and could be facilely conjugated with molecules like biotin. Intriguingly, the PDots showed very low STED power requirement (<3 W for 70 nm resolution) but high photobleaching resistance. In 2020, the potential application of PDots in STED was further explored (Wu et al., 2020). Red and far-red emitting PDots with ∼30 nm size were synthesized and used for immunofluorescence staining of subcellular targets including membrane protein CD44, tubulins, and lysosomes (Figure 5). Furthermore, a dual-color STED imaging was performed to accomplish the real-time tracking of endosome interactions, which suggested the application potential of these materials as long-term, real-time imaging tags for STED microscopy.

### Inorganic Emitters

#### FNDs

FNDs are one of the inorganic nanomaterials initially applied for STED imaging. The PL of these carbon-based nanoemitters comes from defects like nitrogen vacancy centers (NV^−^) or nitrogen-vacancy-nitrogen (N-V-N) in the nano-sized sp^3^ diamond crystals, which endows them with red or green fluorescence (Hsiao et al., 2016). Unlike organic fluorophores, the NV^−^ centers are basically non-photobleaching and nonblinking and have longer PL lifetime (∼20 ns), promoting their STED applications.

The covalent crystalline nature of FNDs guarantees its outstanding optical stability under harsh physical/chemical conditions. However, such characteristics also cause difficulty in morphology/functionalization control during the synthesis (Yu et al., 2005). Typically, the size of synthesized FNDs ranged from 30 to 100 nm, which still hampers its application as a subdiffraction imaging tag to a certain extent.

**FIGURE 6:**
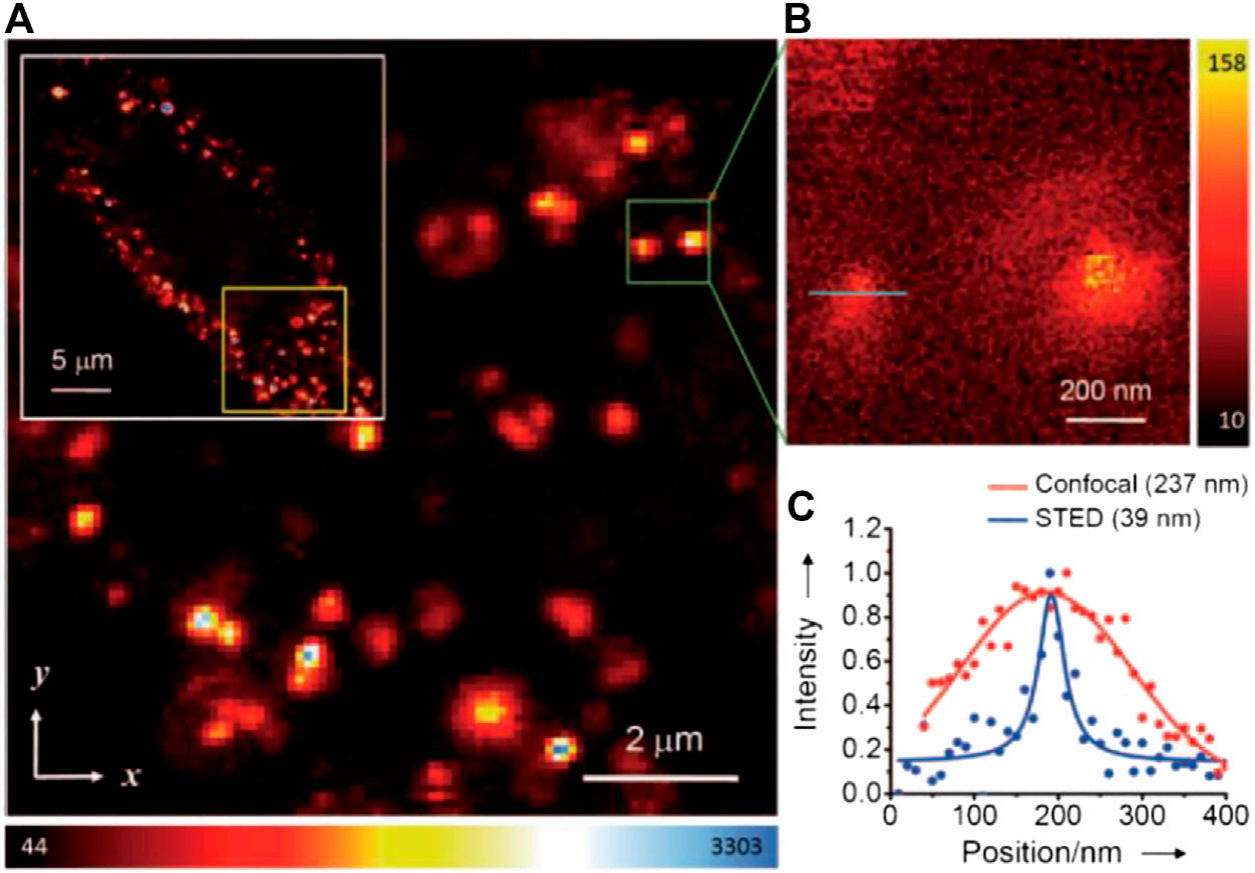
Confocal and STED imaging of HeLa cells labeled with BSA-conjugated FNDs by endocytosis (Tzeng et al., 2011). **(A)** Confocal image of HeLa cells labeled with FNDs. **(B)** STED image of single FNDs. **(C)** Plotfile for confocal and STED imaging results in the region of interest (green line in **B**).

Despite the relatively large particle size of FNDs, the fluorescent NV^−^ centers inside exist at the atomic level, which therefore makes them an ideal target for superresolution imaging. Hell and coworkers first reported the STED imaging with diamond samples with NV^−^ emitting centers with an ultrahigh resolution of ∼6 nm in 2D-STED imaging (Rittweger et al., 2009), a resolution record which was later refreshed to ∼2.4 nm (Wildanger et al., 2012). However, such results can only be obtained with an ultrahigh STED power of several GW/cm^2^. With a reasonably lowered STED power (∼260 mW, considering the endurance of cells), the lateral resolution in STED imaging of dispersed FNDs was ∼40 nm (Han et al., 2009); otherwise, with a 100-mW STED power, the lateral and the axial resolution of 3D-STED imaging could reach ∼100 nm. On this basis, the potential application of FNDs for STED bioimaging was further explored by Chang and coworkers (Tzeng et al., 2011). To overcome the aggregation tendency of FNDs in physiological environments, Chang et al. modified the FNDs nonconvalently with bovine serum albumin (BSA) to improve their delivery efficiency into HeLa cells for nonspecific labeling. Under a STED power of 180 mW, the lateral resolution of individual FNDs in cells reached 39 nm (Figure 6) in accordance with previous results. Similarly, Laporte and Psaltis performed cell imaging with endocytosed green fluorescence FNDs and achieved ∼90 nm resolution with 130 MW/cm^2^ STED intensity (Laporte and Psaltis, 2016).

#### QDots

QDots are nano-sized semiconductor particles with quantum confinement-induced photoluminescent features (Michalet, 2005). Compared with molecular fluorophores, QDots feature higher brightness and photostability, better monochromaticity, and continuously tunable emission (determined by particle size), which altogether facilitate their applications as imaging probes. Despite their composition with heavy metal elements (Cd, Pb, etc.), the toxicity of QDots is still proved acceptable for *in vitro* research (Gao et al., 2005). To this day, the technique of immunofluorescence labeling with QDots has been vastly developed, which facilitates their application for superresolution bioimaging.

In brief, QDots have a series of advantages such as small size (generally <20 nm), high brightness, long PL lifetime, and excellent photostability ensuring its potential in superresolution imaging, especially STED imaging where the photostability of probes is always emphasized. However, the application of QDots in STED also enters some challenges due to the unique optical features of these materials. For example, Auger recombination in isolated QDots is known to suppress the stimulated emission of QDots, which lowers the depletion efficiency by STED, especially in smaller dots (Lesoine et al., 2013). Another issue is that the QDots tend to suffer from re-excitation by the STED light, due to their broad absorption and large two-photon absorption cross section, which could cause high background (Larson, 2003).

In 2013, Lesoine et al. reported the first example of STED imaging of individual QDots. To suppress Auger recombination, Lesoine et al. synthesized QDots with a CdS-coated CdSe structure for enhanced biexciton lifetime (Lesoine et al., 2013). An average 40 ± 10 nm resolution was achieved with a 2.0-nJ STED photon power, which was around 10 times improved compared with the confocal resolution (450 ± 90 nm).

**FIGURE 7:**
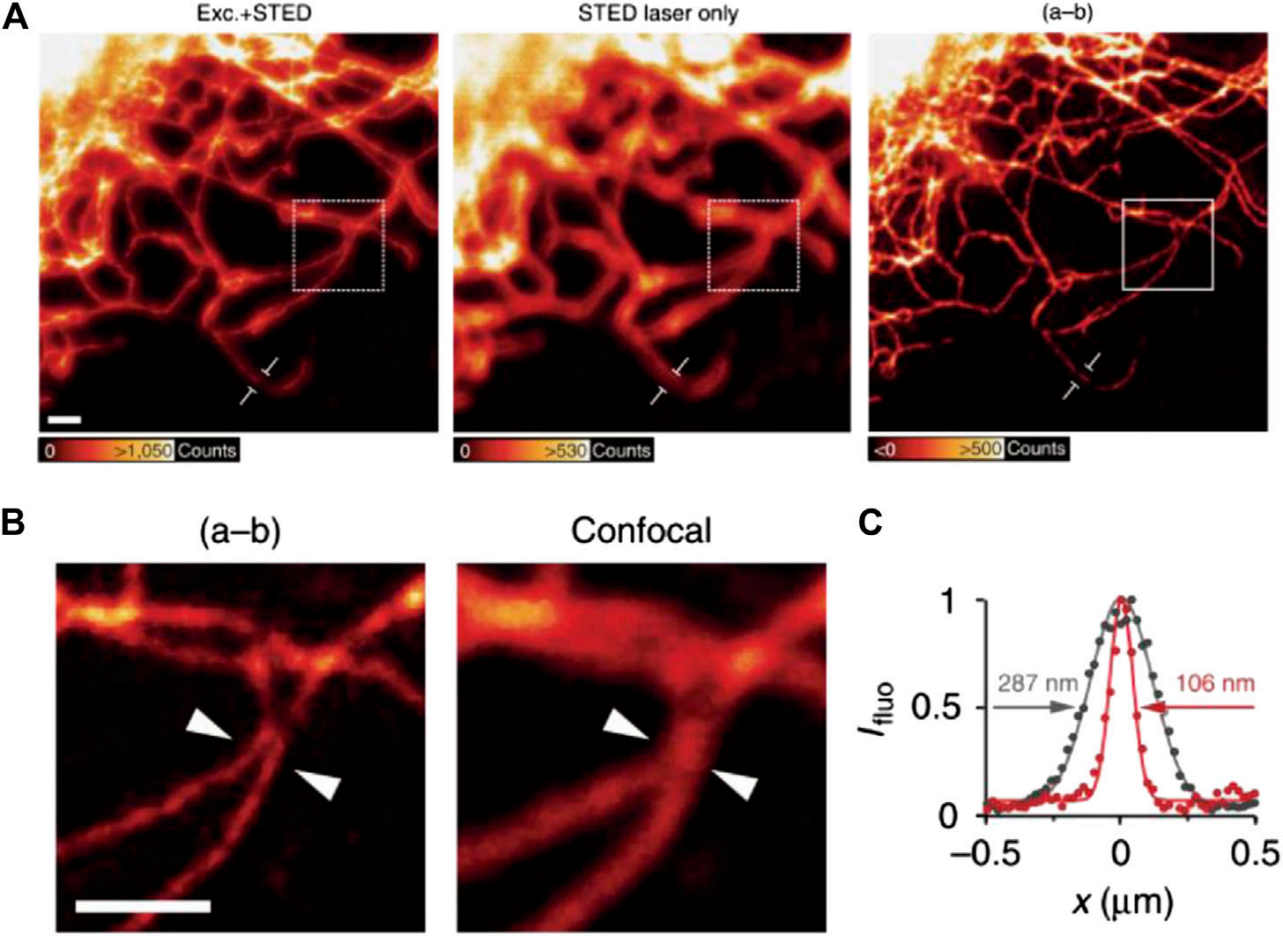
STED imaging of vimentin fibers labeled with commercial QDot705 (Hanne et al., 2015). **(A)** Fluorescence signal acquired by applying excitation + STED, STED light only, and the final image corrected by subtraction. **(B)** The comparison of resolution of corrected STED image and confocal results. **(C)** Plotfile for confocal and corrected STED results in the region of interest.

In 2015, Hanne and Hell et al. reported the first case of STED imaging with a commercially available CdSe QDot: Qdot705 (Hanne et al., 2015). Despite the significant re-excitation (∼26% of the total emission) by 775 nm STED light, subdiffraction resolutions of 54 nm for single particles and ∼100 nm for labeled vimentin fibers were achieved by subtracting the re-excitation background through a second scan (Figure 7).

It should be noted that the improvement in imaging quality and suppression of anti-Stokes emission background could also be accomplished by optimizing the emission and depletion wavelength. Our group recently reported the successful STED imaging of green-emitted CdSe@ZnS QDots with a resolution of 21 nm (Ye et al., 2020) under the excitation/depletion of commercially available 488/592 nm lasers. In contrast to Hanne et al.’s report, the STED laser did not evoke detectable anti-Stokes emission of the QDots in this case, which might be a result of the far separation of spontaneous emission tail and the STED wavelength.

Apart from the group II∼VI semiconductor QDots, the recent-emerging lead-halide perovskite QDots also have shown their potential as STED probes (Ye et al., 2018): in 2018, Ye et al. reported the first example of STED imaging with CsPbBr_3_ QDots. Impressively, a lateral resolution of 20.6 nm was achieved under 39.8 mW STED power.

#### CDots

CDots generally refer to a class of sub-10-nm luminescent particles mostly made of carbon and other nonmetal elements. Since the first discovery of these materials in 2006 (Sun et al., 2006), CDots have long been considered as promising PL probes for biological applications because of their excellent biocompatibility, high photostability (Wang et al., 2014), tunable emission (Ding et al., 2018), and high PLQY (Jia et al., 2019). By virtue of their unique optical and biochemical properties, CDots have found various applications in the fields of bioimaging and theranostics, both for *in vitro* and *in vivo* research.

The first example of CDot-based STED imaging was reported in 2014 by Leménager et al. (2014). Green-emitting CDots with an average diameter of ∼5 nm were incubated with MCF-7 cells, to highlight the lysosomal regions. A resolution of ∼70 nm (>6-fold improvement compared with confocal) was achieved using a commercial Leica SP8 STED confocal microscopy equipped with a 592 nm depletion laser. Pitifully, this pioneer work did not provide much details on the optical performance of CDots under different experimental conditions.

Compared with other nanoprobes, CDots naturally have very small size, which facilitated their transportation into subcellular regions, especially the nucleus. The first attempt to perform nucleus STED imaging with CDots was reported by Han et al. (2019): a green-emitting CDot was synthesized by oxidizing carbon nanoparticles and modified with para-phenylendiamine and 4-carboxybutyl-triphenylphosphonium (PPh^3+^) bromide to obtain a cation surface. The resultant materials are highly affinitive to the negatively charged nucleic acids and showed enhanced emission and red-shifting while binding DNA or RNA molecules. Based on that, two-colored confocal and STED imaging was realized using CDots as the only probe. A *z*-stack 3D reconstructed image of the chromosomes was successfully obtained by 3D-STED imaging without severe photobleaching. However, the depletion efficiency, as well as imaging resolution, was not studied in detail. Meanwhile, Hua et al. developed a strategy to synthesis red-emitting carbon dots for cellular nucleus imaging (Hua et al., 2019). A final resolution of ∼140 nm was obtained in the nucleus region, with a 660 nm STED laser applied for depletion.

**FIGURE 8:**
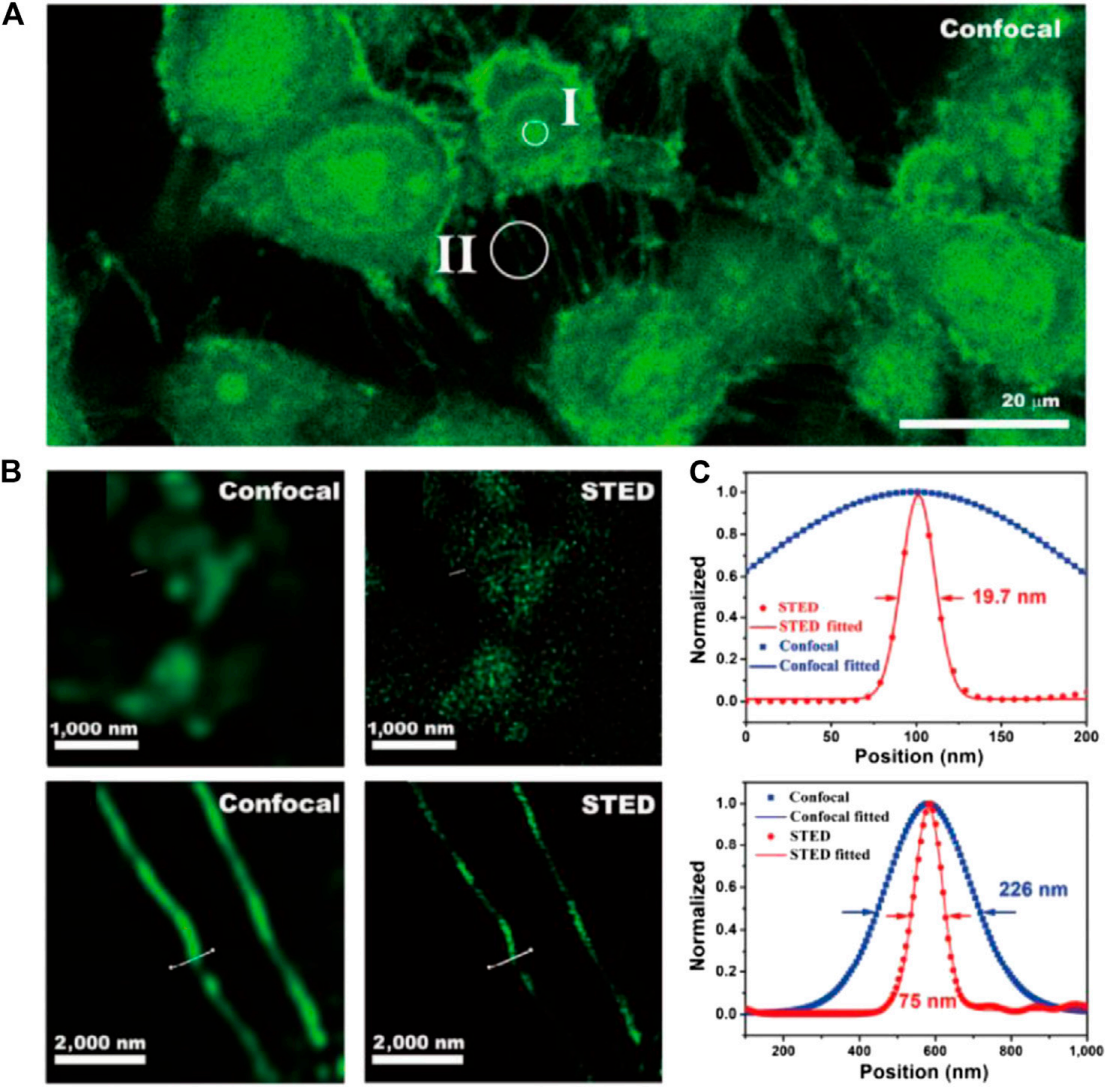
STED imaging of living 4T1 cells stained with CDots (Li et al., 2019). **(A)** Confocal image of 4T1 cells. **(B)** The comparison of STED and confocal images of cells in the nucleus (II) and tunneling nanotubes (II). **(C)** Plotfile for confocal and STED results in both regions of interest.

Despite the unique capability of CDots for nucleus STED imaging, the depletion efficiency and imaging resolution of this material are still under investigation. To this end, our group synthesized F,N-codoped CDots with high PLQY and depletion efficiency (Li et al., 2019). N,F-codoped CDots were synthesized molecular precursors through a one-pot hydrothermal process. The resultant material featured high PLQY (56%), low toxicity, chemical inertness, outstanding photostability, and, above all, high depletion efficiency for STED. Despite the intense emission induced by 592 and 660 nm STED light, these CDs can be efficiently depleted at 775 nm without re-excitation background. The STED resolution of nucleus structure and tunneling nanotubes of CDot-stained 4T1 cells was 19.7 and 75 nm, respectively (Figure 8), and considerably improved compared with confocal imaging results under low STED power (39.6 mW).

Apart from mammal cells, CDs can also be used for the imaging of microorganisms. Yang and coworkers synthesized cation-modified CDots for the labeling of negatively charged *Staphylococcus aureus* bacteria and achieved ∼130 nm subdiffraction resolution under STED imaging condition (Yang J. et al., 2016). More recently, Liu et al. visualized *Streptomyces* *xiamenensis* with internalized CDots, also by adopting a STED imaging setup (Liu S. et al., 2020).

#### UCNPs

UCNPs generally refer to inorganic nanoparticles containing rare-earth elements and dopants (e.g., Yb/Tm-doped NaYF_4_). The luminescence of these materials relies on their multiplex excited state energy influenced by the D and F electron orbits of their metallic component, which combines multiple fascinating characters including the upconverting properties, narrow emission bandwidth, and high photobleaching resistance (Haase and Schäfer, 2011). For decades, the potential bioimaging applications of these materials have been widely explored, taking advantage of their long emission lifetime and near-infrared excitation/emission wavelengths (Chen et al., 2014).

The application of UCNPs for STED-like super-resolved microscopy started in 2010s. Subdiffraction imaging was enabled by manipulating the complex intersystem cross with donut-shaped depletion beams (Kolesov et al., 2011; Wu et al., 2015). However, stimulated emission was not reported in these reports.

**FIGURE 9:**
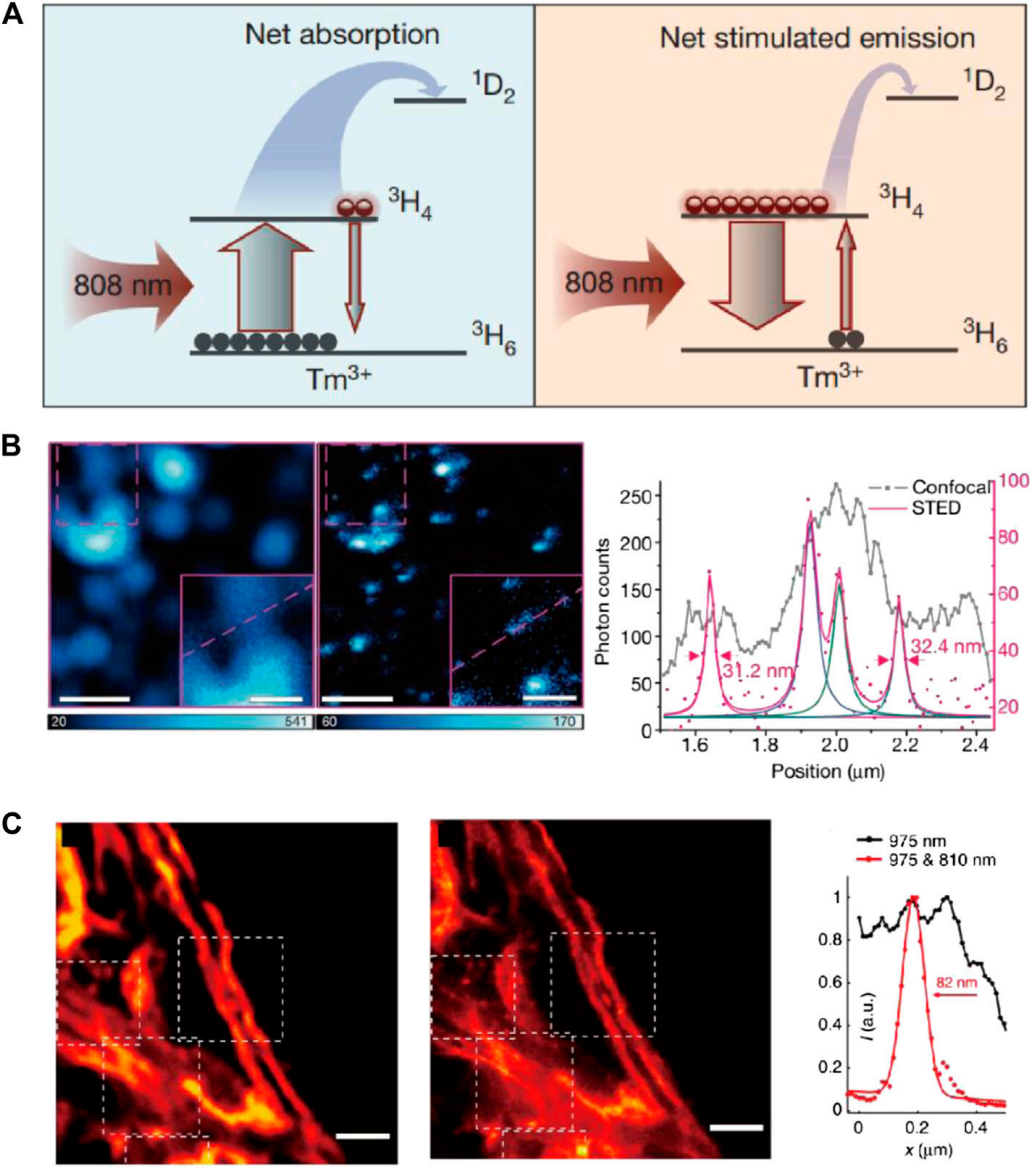
STED performance of UCNPs. **(A)** The diagram of involved energy levels in STED process of Tm-doped UCNPs. **(B)** Particle imaging results and intensity plotfiles of Tm-doped UCNPs with confocal and STED imaging setup (Liu et al., 2017). **(C)** Confocal and STED images of cell skeletons labeled with UCNPs and corresponding plotfiles (Zhan et al., 2017).

The first STED imaging with UCNPs was reported in 2017. Jin and coworkers discovered the amplified stimulated emission in Yb/Tm codoped NaYF_4_ UCNPs and utilized this phenomenon to depopulate the intermediate excited state and deplete the upconverting emission of UCNPs for the first time (Liang and Liu, 2017; Liu et al., 2017). In this case, the Tm^3+^ dopant played an important role in the STED process by introducing the essential intermediate energy states (^3^H_4_ and ^3^H_6_) and enabling the photon-avalanche-like stimulated emission amplification. Unlike most STED setups where the excitation/emission/STED wavelengths each redshift from the left one, the excitation and STED wavelength for UCNPs are shorter than their emission, in order to match the complex energy levels in these inorganic emitters (Figure 9A). For potential bioimaging applications, 13 nm UCNPs were synthesized and imaged with 980 nm excitation and 800 nm depletion light. As a result, a resolution of ∼28 nm (∼1/35 of the excitation wavelength) was reached at 7.5 MW/cm^2^ STED intensity (Figure 9B).

The same year, He, Zhan, and coworkers also independently established results similar to STED imaging with Tm-doped UCNPs (Zhan et al., 2017). He et al. pointed out that the complex cross relaxation in Tm/Tb-doped UCNPs is essential to initiate the STED process, which could be readily enhanced by increasing the content of rare-earth dopants (in accordance with Jin et al.’s conclusion). Different from Jin, He et al. believe the population inversion happened at a higher energy level (^1^D_2_) and assigned the stimulated emission of UCNPs under 810 nm (STED laser) irradiation to the ^1^D_2_→^3^F_2_ transition. A resolution of ∼66 nm was achieved in single-particle STED imaging with 17.7 MW/cm^2^ STED light intensity. For bioimaging applications, the UCNPs were conjugated with antibodies and used for immunofluorescence labeling of cytoskeleton protein in living HeLa cells, which achieved a resolution of 82 nm (Figure 9C).

To further overcome the slow imaging speed [∼4 ms per pixel (Liu et al., 2017)] due to the slow emission transients of UCNPs, Zhan et al. further developed a fast imaging method based on the previous work (Peng et al., 2019). Herein, the content of sensitizer (Yb^3+^) was increased to enhance the emission intensity and accelerate emission kinetics simultaneously, which successfully narrowed down the average pixel dwell time for UCNPs-STED to 10 μs, the same as typical STED scanning speed for molecular probes. Besides the most studied Tm/Yb-doped materials, other UCNPs also showed potential for STED imaging, but with a lower depletion efficiency (Krause et al., 2019): Krause and coworkers tested the STED performance of Dy^3+^- and Eu^3+^-doped NaYF_4_ UCNPs with a resolution of 90 nm at 320 MW/cm^2^ depletion intensity.

Despite the outstanding performance of UCNPs for STED imaging, it should be noted that irremovable re-emission backgrounds occur due to the upconversion excitation, which limits the STED resolution of UCNPs under higher STED laser power. Fortunately, the intensity of such irreversible background amounts to less than 10% of the total emission (Liu et al., 2017; Zhan et al., 2017) and therefore has little influence compared with other issues like particle size and achievable laser power.

#### LPR Hybrids

The LPR effect of noble metal nanoparticles (Au/Ag) has long been adopted as a powerful tool for PL enhancement (Kneipp et al., 1997; Tovmachenko et al., 2006). In 2012, Sivan et al. first established a theoretical research on the possibility of utilizing LPR of metallic nanomaterial to enhance STED imaging quality (Sivan et al., 2012). In this design, the dye was wrapped in a structured gold nanoshell, which had a LPR absorption tuned to the STED wavelength, that is, red-shifted from the emission of dyes. Such structure benefited the STED applications for several reasons: first, the core–shell structure ensured substantial field enhancement in the center, which enhanced the depletion efficiency of the dye by a factor Γ (that is, reduced the required STED power by Γ^−1^). Second, the singlet and triplet excited state decay rates were enhanced due to the metallic shell, which alleviated the photobleaching effect. Third, as a result of the first and second effects, it would be possible to apply higher excitation and depletion power for imaging, leading to improvement in the resolution and signal-to-noise ratio of STED. For demonstration, Sivan and coworkers studied a 26 nm gold particle with 10 nm dye-capsuled silica core as a standard model. The calculated resolution of this nanoparticle-assisted STED method (named NP-STED by Sivan et al.) showed a striking 7 times improvement compared with the conventional STED method. Furthermore, theoretical calculation indicated that the performance of this NP-STED method can be further improved by increasing the thickness of the metallic shell. That being said, NPs with smaller particle size have more potential in realistic applications, considering the intrinsic goal of precise localization in STED imaging. In a following research, Sivan further completed the model of NP-STED with different STED pulse durations (including continuous wave STED), in different time-gating conditions (Sivan, 2012). The abovementioned pioneer works on the concept of NP-STED with plasmon-assisted resolution improvement feature were acknowledged and reviewed by Balzarotti and Stefani, who pointed out that compared with nanoshells, metallic nanorods might be more simple and realistic in practice (Balzarotti and Stefani, 2012). Balzarotti and Stefani envisioned a nanorod particle with two LPR peaks (corresponding to transverse and the longitudinal dipolar modes, respectively) that can be adjusted to amplify both the excitation and STED light by near-field effect. Ideally, the fluorescent probe should be attached near the center of this rod for maximal improvement in the STED resolution and signal-to-noise ratio.

In 2014, applicability of the conceptual NP-STED method was finally supported by solid experimental results (Sonnefraud et al., 2014). Herein, Sonnefraud, Sivan, and other coworkers managed to synthesize batches of uniformed hybrid nanoparticles with 60 nm fluorescent cores and 20-nm-thick gold shells. This composite gives a 670-nm emission peak and a 780-nm LPR absorption, the latter of which efficiently overlapped with the STED wavelength. In this case, the depletion efficiency was enhanced by ∼4 times (Γ = 3.8 ± 0.8). The reason for the small Γ value might be due to the existence of background fluorescence of metallic components, which was not considered in the theoretical model. In the experimental work by Cortés, Sivan, and other coworkers, they further improved the results of NP-STED by using gold nanorods instead of shell structures (Cortés et al., 2016). Compared with the former strategy, conjugating dyes onto the rods avoided unwanted quenching of fluorophors nearby the metal, and the overall metal usage was also reduced owing to the more effective LPR effect. As a result, the enhancement of STED can be further facilitated, and the parasitic background of gold luminescence clearly reduced. The confluence of multiple positive effects gives a doubled Γ value of ∼8.5. However, the final imaging resolution of two studies for dispersed particles is only slightly higher than that of the confocal diffraction limits (∼200 nm).

**FIGURE 10:**
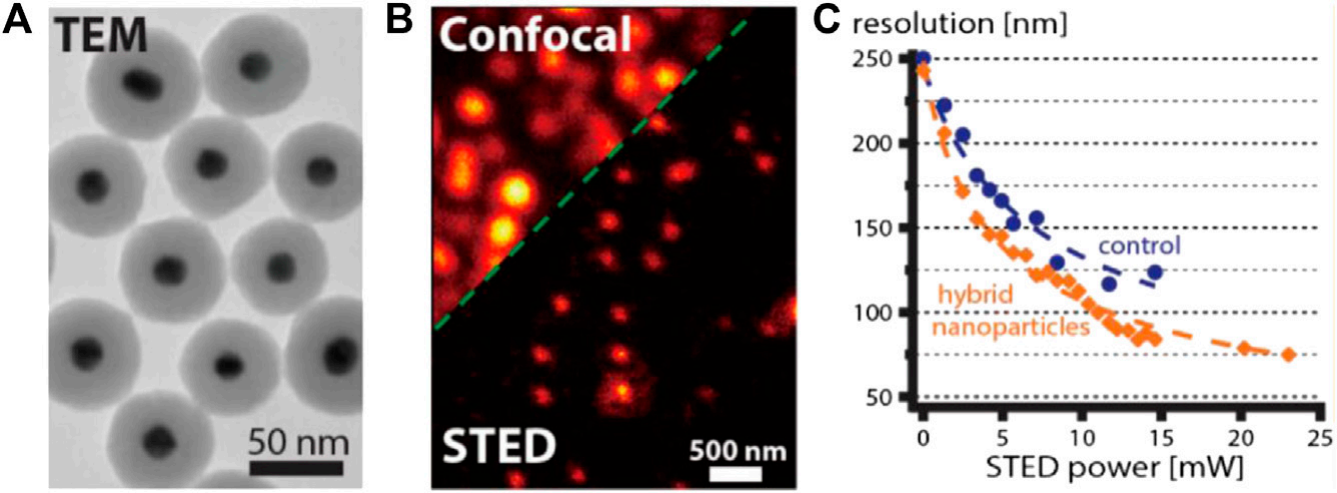
STED performance of typical LPR hybrids (Urban et al., 2018). **(A)** The transmission electronic microscopy (TEM) image of LPR hybrids synthesized by Urban et al. **(B)** Confocal and STED imaging results of single LPR hybrid particles. **(C)** The comparison of resolution enhancement for dye-doped particles with and without (control) LPR effect under different STED powers.

Clearly, further improvement in resolution is required to promote realistic applications of NP-STED in bioimaging. To this end, Hell and Sivan et al. further developed 50 nm LPR hybrid particles with gold core and a silica shell doped with molecular dye Atto488. Such structure led to a variable-field enhancement effect within the particle that decayed as the distance from metallic core increased. As a result, the overall near-field enhancement level is expected to be lower than that of the gold shell or nanorod particles. Interestingly, despite the lower Γ value predicted by calculation (∼1.34 for average particles), the overall performance of this new material actually exceeded the previously reported ones (Urban et al., 2018). Specifically, a final resolution of 75 nm was achieved with a STED power of ∼20 mW (Figure 10), which equaled to a 3.3-fold improvement in resolution with respect to the diffraction limit of confocal microscopy. Meanwhile, the power requirement was ∼1.75 times lower than that needed for the standard dye (8.1∼10 MW/cm^2^).

**FIGURE 11:**
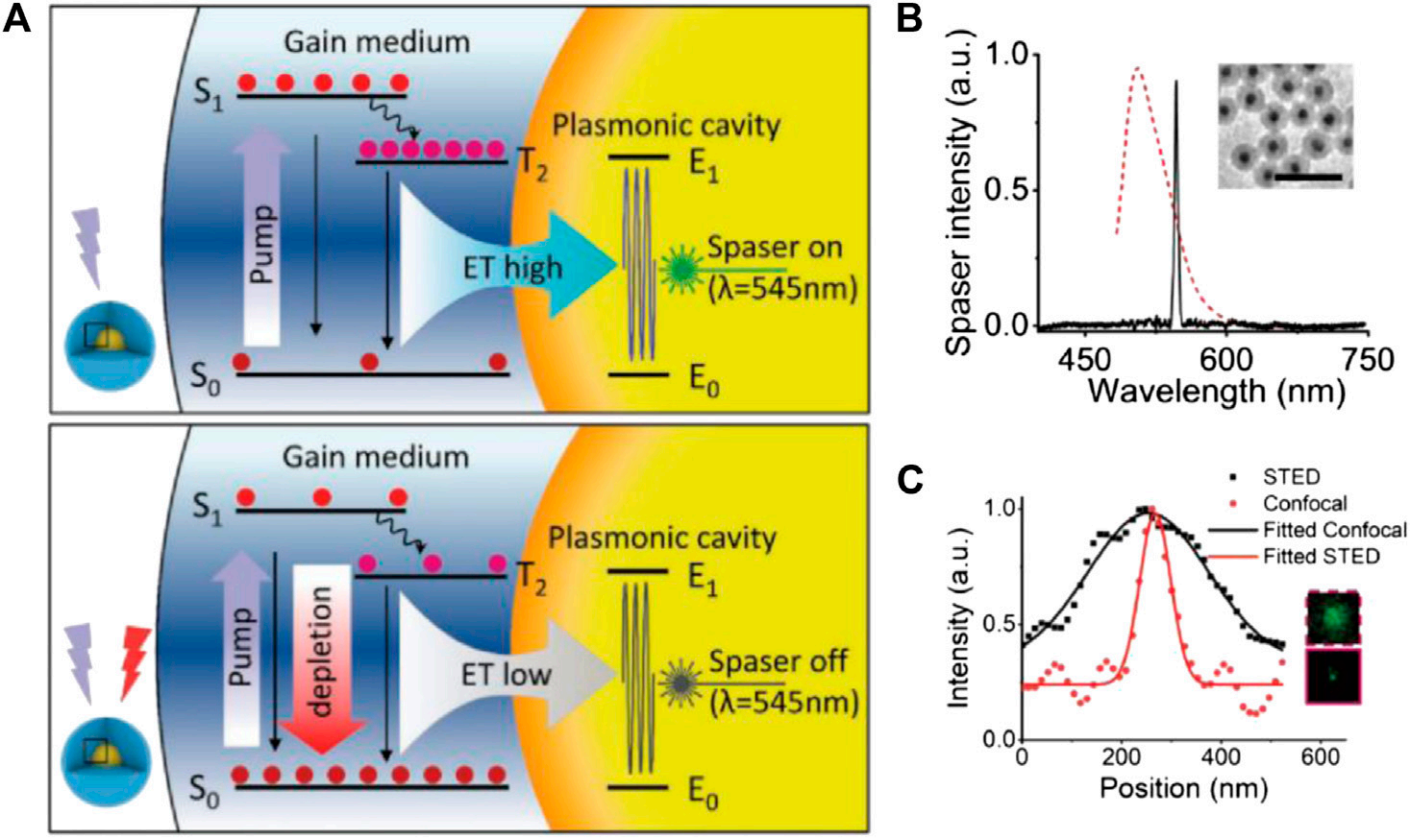
STED performance of spaser nanoprobe (Gao et al., 2020). **(A)** The energy diagram of spaser in STED; notice that the energy of STED light is higher than the spontaneous emission energy. **(B)** Typical emission spectra of spaser (black solid) and the original dye (red dotted). **(C)** Resolution enhancement from confocal to STED imaging and the corresponding plot files.

It is also worth noting that the LPR field enhancement effect is a versatile tool that not only amplifies STED effect but also modulates the excitation-emission dynamics and, in some cases, entirely alters the characteristics of the original spontaneous fluorescence. A good example was given by the surface plasmon laser (spaser) technique, in which surface plasmon of noble metals was used to induce the lasing emission of fluorophores in hybrid nanoparticles (Galanzha et al., 2017). The resultant emissions are highly efficient, monochromatic, and stable, which all benefit their potential bioimaging applications. Recently, Kang and coworkers demonstrated the first example of spaser-based STED (Gao et al., 2020). Similar to the conventional STED process, the depletion of spaser emission power under increasing STED laser power corresponds well with the typical square root law. However, the role of metallic plasmon in this case is quite different from that of the NP-STED model. As depicted in Figure 11, the plasmonic cavity facilitates the spaser emission from the T2 energy level, while the depletion by STED happened on the S1 level (which corresponds to the spontaneous fluorescence of the bare dyes without spaser construction). Such configuration is quite the opposite of most STED processes but yields similar resolution improvement. With a 300-mW STED power, the spaser probe proved a ∼74-nm resolution, together with an ultranarrow emission bandwidth of ∼10 nm.

## Pros and Cons of STED Nanoprobes: Systematic Comparison

To further illustrate the advantages and disadvantages of different STED nanoprobes, herein we systematically cross-compare these materials with molecular STED probes in terms of morphology and functionalization, optical performance, and STED bioimaging utility.

### Morphology and Functionalization

#### Particle Size

**FIGURE 12:**
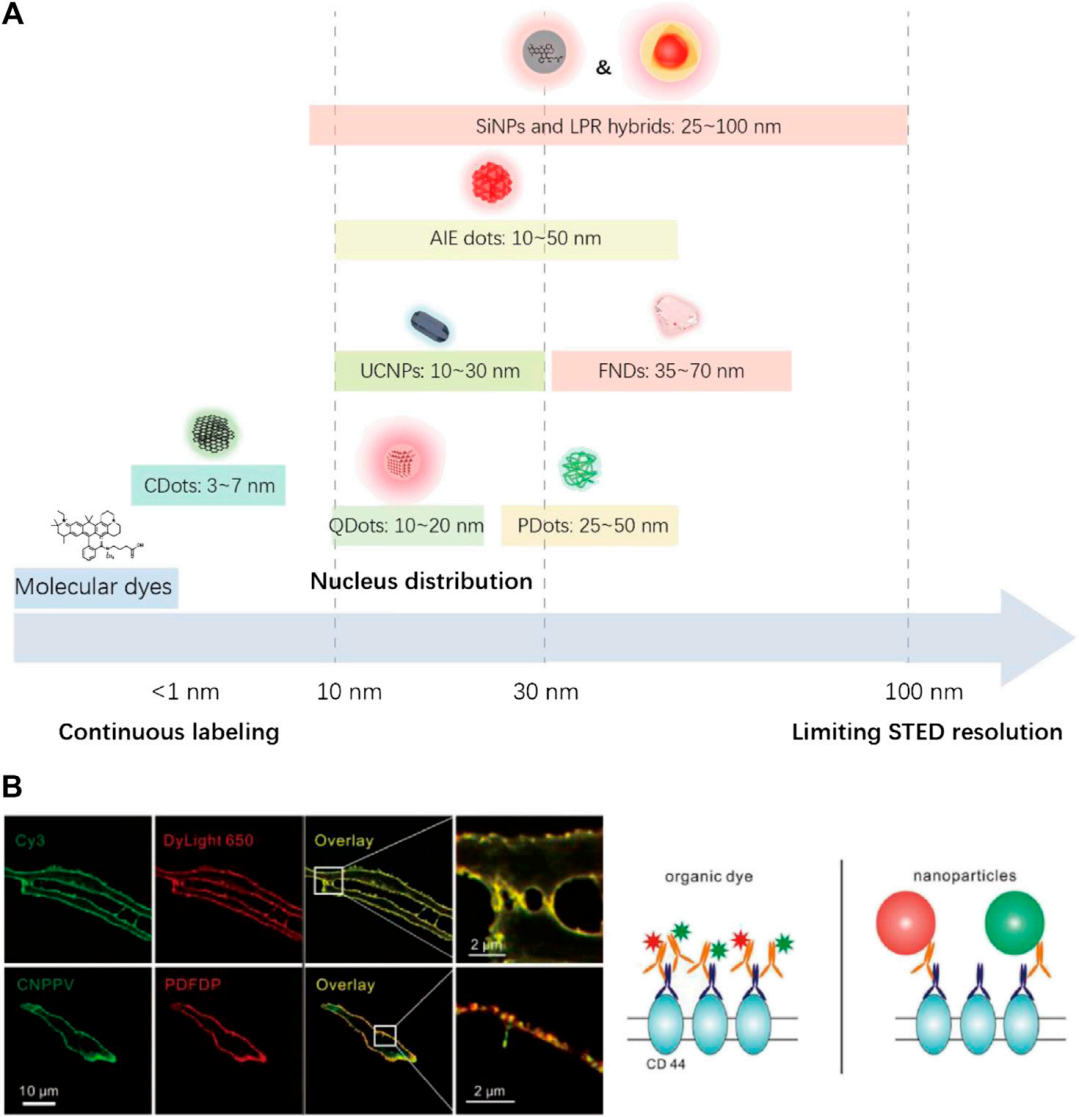
**(A)** Size distribution of reported STED nanoprobes (also see Table 1). **(B)** The observation of discontinuous labeling of nanoparticles due to steric effect (Wu et al., 2020).

The currently reported STED nanoprobes have different sizes ranging from ∼2 nm to slightly over 100 nm (see Table 1; Figure 12A). The larger size of these materials compared with molecular probes (mostly <1 nm) raised several issues, which may interfere with the STED imaging quality. For example, the labeling density of nanoparticles is inevitably lower than that of molecules, due to their larger size (Figure 12B; Wu et al., 2020). Furthermore, nanoparticles beyond ∼30 nm suffer from insufficient transportation into and within cells, which might lead to poor targeting ability in bioimaging (Schübbe et al., 2012). Particles with even a larger size might induce an unneglectable steric effect, which severely interferes with subcellular targeting and may cause potential cellular damage, and meanwhile also put a physical limitation on the resolution (Schubbe et al., 2010; Prabhakar et al., 2013).

**TABLE 1:** Summary of the particle sizes and surface functionalizing methods for STED probes.

Categories	Size	Functionalizing methods	References
Molecular dyes and FPs	<1 nm		Hein et al. (2008), Meyer et al. (2008), Wildanger et al. (2009)
Dye-doped SiNPs	Tunable from 30 to 100 nm; smallest size <2 nm	Through silane linkers	Schubbe et al. (2010), Schübbe et al. (2012), Peuschel et al. (2015), Tavernaro et al. (2017), Man et al. (2019), Liang et al. (2020)
AIE dots	10∼50 nm	Amidation (NHS-EDC)	Yu et al. (2015), Fang et al. (2017), Li et al. (2017), Li et al. (2018), Xu et al. (2020)
PDots	25∼50 nm	Amidation (NHS-EDC)	Wu et al. (2018b), Wu et al. (2020)
FNDs	35∼70 nm	Noncovalent passivation	Han et al. (2009), Rittweger et al. (2009), Tzeng et al. (2011), Prabhakar et al. (2013), Laporte and Psaltis, (2016)
QDots	10∼20 nm	Ligands + amidation (NHS-EDC)	Lesoine et al. (2013), Hanne et al. (2015), Yang et al. (2016b), Ye et al. (2018), Ye et al. (2020)
CDots	3∼7 nm	Amidation (NHS-EDC/SOCl_2_)	Leménager et al. (2014), Yang et al. (2016a), Han et al. (2019), Hua et al. (2019), Li et al. (2019), Liu et al. (2020b)
UCNPs	10∼30 nm	Ligand + amidation (NHS-EDC)	Liu et al. (2017), Zhan et al. (2017), Peng et al. (2019)
LPR hybrids	25∼100 nm	Streptavidin–biotin binding	Sonnefraud et al. (2014), Cortés et al. (2016), Urban et al. (2018), Gao et al. (2020)

Fortunately, the size control method of most colloidal materials is mature nowadays, and sub-30-nm-sized NPs, including UCNPs (Liu et al., 2017; Zhan et al., 2017), SiNPs (Liang et al., 2020), AIE dots (Fang et al., 2017; Li et al., 2017; Li et al., 2018), PDots (Wu et al., 2020), and QDots (Lesoine et al., 2013; Hanne et al., 2015; Ye et al., 2018; Zhao et al., 2019), have all been successfully fabricated for STED applications, some of which are even commercially available (Hanne et al., 2015; Zhao et al., 2019). CDots (Leménager et al., 2014; Wang et al., 2017; Han et al., 2019; Hua et al., 2019; Li et al., 2019), on the contrary, are naturally nano-sized (<10 nm) and are well known for their fast transport dynamics across membranes (Lu et al., 2019). For FNDs and LPR nanohybrid particles, the smallest particle size in reported STED imaging cases is 40∼50 nm (Tzeng et al., 2011; Urban et al., 2018). However, such limitations could be overcome, as the synthesis of smaller particles has also been reported (Liu P. et al., 2020; Ma et al., 2020).

In this sense, despite a few cases, the disadvantages of large particle size have been well addressed for most STED nanoprobes. Subsequently, synthesizing sub-5-nm-sized nanoparticles (Hua et al., 2019; Liang et al., 2020) should be the next target, as they in principle could provide a better biological and optical performance without losing the ultrafine targeting ability of molecular probes.

#### Functionalizing Methods

Compared with molecular dyes or FPs, the modification of nanoparticles faces more challenges due to significant steric effects. Still, many applicable functionalizing methods have been put forward, and some have proved applicable for the modification of STED nanoprobes (see Table 1).

Nanoparticles with full organic composition (AIE dots and PDots) usually consist of highly carboxylated surficial structures (Fang et al., 2017; Wu Y. et al., 2018; Wu et al., 2020). Such characters enable the facile functionalization of these particles with aminated molecules or proteins through the highly efficient amidation catalyzed by N-hydroxysuccinimide (NHS) and 1-(3-dimethylaminopropyl)-3-ethylcarbodiimide (EDC). For carbon materials like FNDs or CDots, surficial carboxyl groups can be induced by chemical oxidation (Tzeng et al., 2011; Han et al., 2019). Despite that, FNDs still suffer from severe aggregation tendency and have only been successfully passivated by a noncovalent method, due to their large size (Tzeng et al., 2011). Comparatively, nano-sized CDots can be more easily functionalized by chemical methods, utilizing either their carboxyl (Han et al., 2019) or amine groups for modification (Yang J. et al., 2016). Such strategies have been adopted to produce CDot probes for the targeting of different organelles in living cells (Wu X. et al., 2018), which might play important roles in STED imaging.

By contrast, nanoprobes with inorganic surface undergo more complicated functionalization routes. For SiNPs, their surficial Si-OH groups are not very reactive with most linkage groups (carboxyl, amine, etc.). Therefore, the functionalization of SiNPs is mostly performed through silane linkers like APTES before further modification with functional molecules by amidation (Schubbe et al., 2010; Schübbe et al., 2012; Man et al., 2019). This might lead to an increase in particle size during the modification, altering the biodistribution of these probes, especially for smaller particles.

Inorganic emitters with metal element on their surface (QDots and UCNPs) can be functionalized with ligands that introduce active functional groups, which further enable the fabrication of bioconjugates for immunofluorescence label (Hanne et al., 2015; Zhan et al., 2017). At present, such strategies have been fully developed and utilized in commercially available QD probes. Meanwhile, gold-shelled LPR hybrids can be directly modified with streptavidin and further bound with biotinylated structures (Cortés et al., 2016).

### Optical Performance

The optical performance of different nanoparticles can be compared in the following different dimensions (see Table 2).

**TABLE 2:** Summary of photostability and saturation intensities for STED probes.

Categories	Photostability	*I*_*sat*_	References
Molecular dyes and FPs	Very poor, bleachable within tens of STED scans	10^1∼2^ MW/cm^2^	Hein et al. (2008), Meyer et al. (2008), Wildanger et al. (2009)
Dye-doped SiNPs	Poor [<50% intensity remains after 15 min of continuous scanning; exception: (Man et al. (2019)]	∼10^–1∼2^ MW/cm^2^ depending on dyes	Schubbe et al. (2010), Schübbe et al. (2012), Peuschel et al. (2015), Tavernaro et al. (2017), Man et al. (2019), Liang et al. (2020)
		Lowest reported value: 0.18∼0.188 MW/cm^2^ (Man et al. (2019), Liang et al. (2020)	
AIE dots	Robust (>50% intensity remains after 30 min of continuous STED scanning)	∼10^1∼2^ MW/cm^2^ (estimated)	Yu et al. (2015), Fang et al. (2017), Li et al. (2017), Li et al. (2018), Xu et al., 2020)
PDots	Robust (>50% intensity remains after 2 h of continuous STED scanning)	∼10^–1^ MW/cm^2^ (estimated)	Wu et al. (2018b), Wu et al. (2020)
FNDs	Non-photoleaching	0.7∼6.6 MW/cm^2^	Han et al. (2009), Rittweger et al. (2009), Tzeng et al. (2011), Prabhakar et al. (2013), Laporte and Psaltis, (2016)
QDots	Robust (>50% intensity remains after 2∼3 h of continuous STED scanning or thousands of scans)	0.129∼0.192 MW/cm^2^	Lesoine et al. (2013), Hanne et al. (2015), Yang et al. (2016b), Ye et al. (2018), Ye et al. (2020)
CDots	Robust (no significant bleaching after ∼1000 continuous scans)	0.226 MW/cm^2^ Li et al. (2019)	Leménager et al. (2014), Yang et al. (2016a), Han et al. (2019), Hua et al. (2019), Li et al. (2019), Liu et al. (2020b)
UCNPs	Robust (no significant bleaching after ∼200 min of continuous STED scaning)	0.19∼0.849 MW/cm^2^	Liu et al. (2017), Zhan et al. (2017), Peng et al. (2019)
LPR hybrids	Improved compared with molecular dyes or SiNPs	4.6∼5.8 MW/cm^2^	(Sonnefraud et al. (2014), Cortés et al. (2016), Urban et al. (2018), Gao et al. (2020)

### Photostability

Generally speaking, nanoparticle probes, compared with molecular probes, are more stable against photobleaching, which benefits their applications in long-term and 3D-STED imaging (Han et al., 2009; Ye et al., 2018; Han et al., 2019). The increased photostability of nanoparticles can be explained in several ways. First, the compact nanostructure of these materials separated the photoluimincent centers from environmental oxygen, thus effectively alleviating the photo-oxidation effect, which causes permanent bleaching of fluorophores. Second, the compact fluorophores and/or solid media provide additional intramolecular relaxation pathways, which also help alleviate photobleaching.

Inorganic emitters like QDots, UCNPs, and FNDs perform well under intense irradiation (×10^1∼2^ MW/cm^2^) for ∼1 h (Han et al., 2009; Rittweger et al., 2009; Lesoine et al., 2013; Hanne et al., 2015; Laporte and Psaltis, 2016; Zhan et al., 2017; Ye et al., 2020), due to the high stability of inorganic crystalline structures compared with linear covalent bonds. Specifically, FNDs are highly stable to extremely high STED power (several GW/cm^2^), which allows nanoscale STED resolution comparable to electronic microscopy (Rittweger et al., 2009; Wildanger et al., 2012).

CDots are usually considered as highly photostable materials that endure tens of MW/cm^2^ STED power (Leménager et al., 2014; Wang et al., 2014; Li et al., 2019), but there are also evidence that some of the CDots are not as stable as typical inorganic nanomaterials (still, more stable than molecular fluorophores) (Hua et al., 2019), which suggests that their fluorescence might be related to embedded fluorophores rather than crystalline carbon cores (Qu and Sun, 2020).

Condensed organic nanoparticles like AIE dots and PDots are also resistant to photobleaching and can typically perform continuous STED imaging for up to 1∼2 h with over 50% fluorescence intensities remaining (Li et al., 2017; Wu Y. et al., 2018; Wu et al., 2020). Compared with other nanoparticles, dye-doped SiNPs suffer most from photobleaching, as their fluorescence originates from dispersed molecular emitters (Tavernaro et al., 2017). This issue can be addressed by either introducing metallic LPR structures (Sivan et al., 2012) or eliminating SOC by material engineering (Liang et al., 2020), which enhances their photostability to an acceptable level, similar to other nanoparticles.

### Saturation Intensity

Saturation intensity is another crucial property of STED nanoprobes. In a typical STED imaging setup, the resolution is given as

$$\delta_{\mathrm{STED}}=\frac{\lambda }{2\mathrm{NA}\sqrt{\mathrm{1+}I_{\mathrm{STED}}/I_{\mathrm{sat}}}}$$ (1)

where *I*_*STED*_ and *I*_*sat*_ are the applied STED power and the saturation power of the materials, respectively. Considering the resolution limit for confocal imaging,

$$\delta_{\mathrm{confocal}}=\frac{\lambda }{2\mathrm{NA}}$$ (2)

For most molecular and protein STED probes, their *I*_*sat*_ values range from 10^1∼2^ MW/cm^2^ (Hein et al., 2008; Bianchini et al., 2012). In this sense, the STED power required to achieve ∼100 nm resolution (approximately 2∼4 times improved from the limiting resolution of confocal imaging) easily exceeded 100 MW/cm^2^, which causes potential cellular damage and severe photobleaching. Apparently, lowering the *I*_*sat*_ value could help reducing the STED power requirement and improve the STED imaging quality. According to Schrof and Hell et al. (Schrof et al., 2011), the saturation intensity of a STED probe is determined by

$$I_{\mathrm{sat}}=\frac{\mathrm{h\nu}}{\sigma \tau }$$ (3)

with *hν* being the photon energy, σ the cross section for stimulated emission, and τ the PL lifetime. Compared with the dispersed molecular emitters, the compact nanoemitters typically show higher brightness and larger optical cross sections, which contribute to a lower saturation intensity or, equally, higher depletion efficiency. Furthermore, inorganic emitters like UCNPs and QDots typically have a longer PL lifetime (×10 ns), which also contribute to their low saturation intensity values. To this day, nanoprobes like UCNPs (Liu et al., 2017; Liang and Liu, 2017), QDots (Ye et al., 2020; Ye et al., 2018), CDots (Li et al., 2019), and SiNPs (Liang et al., 2020; Man et al., 2019) have been reported with an ultralow saturation intensity of 10^–1^ MW/cm^2^, which are 2∼3 magnitude lower than that of typical molecular STED probes. For FNDs, the lowest reported saturation intensity for N-V and N-V-N defects was 1 MW/cm^2^ (Han et al., 2009) and 2.5 MW/cm^2^ (Laporte and Psaltis, 2016), respectively, slightly lower than that of molecular probes.

It should be noted that compared with other materials, the saturation intensities of SiNPs may vary a lot according to the specific type of dyes used for doping. For example, SiNPs doped with the Atto647N dye showed STED power requirement (100∼400 MW/cm^2^) similar to that of the bare dyes (Tavernaro et al., 2017). Meanwhile, SiNPs doped with specially designed fluorophores (non-SOC (Liang et al., 2020) or non-ACQ (Man et al., 2019)) showed an ultralow *I*_*sat*_ of ∼10^–1^ MW/cm^2^.

The exact saturation intensity values of AIE dots and PDots are absent from the literature, which can be estimated according to their power requirement for STED imaging. For AIE dots, their saturation intensities are estimated to be in the range of 10^1∼2^ MW/cm^2^, judged from both the STED power requirement for imaging (>100 mW) (Fang et al., 2017; Li et al., 2017; Li et al., 2018; Xu et al., 2020) and the comparison of their depletion efficiency with molecular probes like coumarin (Yu et al., 2015). Although their depletion efficiency is similar to that of molecular dyes, the achievable resolution has been improved (Li et al., 2017) due to their higher endurance to STED photons. For PDots, the saturation intensities are estimated to be ∼10^–1^ MW/cm^2^.

LPR hybrids have smaller saturation intensity values than the original dyes used in the hybrid, which are determined by

$$I_{\mathrm{sat’}}=\frac{I_{\mathrm{sat}}}{\Gamma }$$ (4)

where *I*_*sat’*_ is the effective saturation intensity of LPR hybrid, *I*_*sat*_ is the saturation intensity value of the dyes, and Γ is the factor describing the enhancement of depletion effect induced by LPR. So far, the reported *I*_*sat’*_ of LPR hybrids is still limited to ∼5 MW/cm^2^ (Sonnefraud et al., 2014; Urban et al., 2018), partially due to the high *I*_*sat*_ value of the molecular dyes used in the hybrids.

### Excitation/Emission Features

Basic excitation/emission features of the probes, such as PLQY, fluorescence lifetime, and potential re-excitation, play important roles in the STED bioimaging.

PLQY values indicate how efficient the fluorescent probe converts excitation light into emission signals. Probes with higher PLQY may provide a better signal-to-noise ratio in imaging similar excitation and depletion conditions. Herein, the reported PLQY values of different nanoprobes for STED imaging are summarized, as shown in Table 3. FNDs and QDs showed highest average quantum yield above ∼70%, followed by CDots, PDots, and AIE dots, whose PLQY varied between 10 and 60% depending on the specific materials. The PLQY of SiNPs and LPR hybrids is highly dependent on the doped dyes and may reach a near-unity level with optimized condition (Liang et al., 2020). As for UCNPs, although their PLQY was not provided in relative research, the values are estimated to be <5% according to relative research on similar materials (Chen et al., 2012). The relative low-emitting efficiency however can be compensated by applying higher excitation power [1 mW (Liu et al., 2017) or 700 kW/cm^2^ (Zhan et al., 2017)], which has been performed in STED imaging.

**TABLE 3:** Summary of excitation/emission features for different STED nanoprobes.

Categories	PLQY	Lifetime	Pixel dwell times	Re-excitation	References
Dye-doped SiNPs	Depending on dyes, up to 99% Liang et al. (2020)	1∼10 ns	∼10 μs	Not mentioned	Schubbe et al. (2010), Schübbe et al. (2012), Peuschel et al. (2015), Tavernaro et al. (2017), Man et al. (2019), Liang et al. (2020)
AIE dots	20∼30%	1∼5 ns	Not mentioned	No in most cases (exception: Li et al. (2017))	Yu et al. (2015), Fang et al. (2017), Li et al. (2017), Li et al. (2018), Xu et al. (2020)
PDots	20∼50%	\	0.5∼1 ms	No	Wu et al. (2018b), Wu et al. (2020)
FNDs	70∼95%	∼12 ns (N-V defects)	1∼10 ms	No	Han et al. (2009), Rittweger et al. (2009), Tzeng et al. (2011), Prabhakar et al. (2013), Laporte and Psaltis, (2016)
		/27 ns (N-V-N defects)			
QDots	64∼90.5%	8∼10 ns	10∼100 μs	Yes (3∼26%)	Lesoine et al. (2013), Hanne et al. (2015), Yang et al. (2016b), Ye et al. (2018), Ye et al. (2020)
				Can be avoided	
CDots	14.5∼56%	∼5 ns	Not mentioned	Yes, can be avoided	Leménager et al. (2014), Yang et al. (2016a), Han et al. (2019), Hua et al. (2019), Li et al. (2019), Liu et al. (2020b)
UCNPs	\	∼10^0∼1^ μs	Typically, 1∼10 ms. shortest reported value: 10 μs Peng et al. (2019)	Yes (<10%)	Liu et al. (2017), Zhan et al. (2017), Peng et al. (2019)
LPR hybrids	\	∼0.9 ns	10∼100 μs	Yes	Sonnefraud et al. (2014), Cortés et al. (2016), Urban et al. (2018), Gao et al. (2020)

In terms of PL lifetime, most STED nanoprobes including organic emitters (AIE dots, PDots, and dispersed dye molecules) (Li et al., 2017; Liang et al., 2020), FNDs (Rittweger et al., 2009), QDots (Ye et al., 2020), and CDots (Li et al., 2019) have short PL lifetime ranging from 1 to 20 ns, which allows fast scanning speed and similar time-gating setup similar to molecular probes for the improvement in STED imaging quality (Wang et al., 2018). The pixel dwell times of these nanoprobes are therefore typically shorter than 1 ms. The only exception was FNDs (1∼10 ms per pixel), which required longer pixel dwell times to compensate for the low signal intensities under extreme STED power. UCNPs however have much longer lifetimes scale up to μs due to complex intersystem relaxation (Liu et al., 2017), which leads to a much longer dwell time per pixel (typically 1∼10 ms) than other probes. Although increasing the content of sensitizer ion may accelerate the emission kinetics (Peng et al., 2019), it might also increase the power requirement for STED imaging, as suggested in Eq. 3.

The re-excitation in STED imaging refers to a situation where the STED light alone induces unneglectable emission of the probes. In this case, a parasitic background fluorescence always exists and even enhances in the donut-shaped STED light irradiated region, which leads to dim halos in the image and prevent the further improvement in resolution (Figure 7). So far, nanoprobes including QDots, CDots, UCNPs, and LPR hybrids have witness re-excitation in their STED applications (see Table 3). For QDots, the re-excitation intensity might reach up to 26% of the total emission, which clearly damaged the imaging resolution and signal-to-noise ratio (Hanne et al., 2015). This issue however can be facilely addressed by rationally selecting a longer STED wavelength located apart from the tail of QDots’ emission (Ye et al., 2020). Similarly, the re-excitation of CDots could also be avoided by using STED light with a longer wavelength (Li et al., 2019). The re-excitation of UCNPs, on the other hand, is difficult to avoid, due to their multiplex energy levels that pick up anti-Stokes excitations easily. Luckily, the re-excitation observed in UCNPs is not very intense (<10% as reported in (Liu et al., 2017) and <4% as reported in (Zhan et al., 2017)) and therefore can be omitted during the imaging. The re-excitation of LPR hybrids originates from their metallic fluorescence, which can be distinguished from the fluorescence of doped dyes by time-gating (Sonnefraud et al., 2014). Meanwhile, PDots, AIE dots, and FNDs are mostly free from re-excitation due to their relatively large Stokes shift (>100 nm), apart from occasional exceptions (Li et al., 2017).

Besides the careful selection of STED wavelength, the re-excitation can also be eliminated by other experimental or instrumental methods. For example, by applying STEDD (stimulated emission double depletion) imaging with two depletion pulses, the re-excitation background can be effectively subtracted (Gao and Ulrich Nienhaus, 2017). In other situations, the anti-Stokes emission background evoked by depletion laser generally has a shorter PL lifetime compared with the down-converted fluorescence, which therefore allows time-related background subtraction by gated STED or STED-FLIM technology (Wang et al., 2018; Ma and Ha, 2019). Another known solution is to apply adaptive STED illumination, aka the DyMIN (Dynamic intensity MINimum) method, which not only eliminates re-excitation but also alleviates photobleaching at the same time (Li et al., 2020). Still, the abovementioned methods pose high instrumental requirements that might not be satisfied by most STED devices.

### STED Bioimaging Utility

The actual STED bioimaging utility of nanoparticles is determined by multiple factors, including the biocompatibility, targeting, real-time tracking ability, and power requirement.

### Biocompatibility

To meet the requirements of bioimaging, especially for living cells and long-term applications, the nanoprobes must be biocompatible and nontoxic in usage. The cytotoxicity of all nanoprobes applied for STED has been extensively studied (Soenen et al., 2011; Luo et al., 2013; Wolfbeis, 2015; Reisch and Klymchenko, 2016). The conclusion can be summarized as follows:

In terms of chemical composition, materials like SiNPs, AIE dots, PDots, FNDs, and CDots that consist of nonmetal elements are generally biocompatible and nearly nontoxic with a concentration of several tens of μg/mL, which is normally 5∼10 times higher than the working concentration used in imaging. Still, it should be noted that materials with large size (∼100 nm) and/or aggregation tendency might induce cellular damage in long-term studies. This particularly limits the usage of bare SiNPs and FNDs, as their surface composition is hydrophobic and might form agglomerates under physiological conditions (Schubbe et al., 2010; Tzeng et al., 2011). Meanwhile, inorganic nanoparticles like QDots, UCNPs, and LPR hybrids contain heavy metal elements, resulting in potential long-term toxicity, especially for *in vivo* imaging. Fortunately, since the toxicity of inorganic nanoparticles can be largely alleviated by bioconjugation and the dosages required for imaging are generally low, the toxicity of these materials for cellular imaging has proved acceptable (Gao et al., 2005).

Another important issue however is the phototoxicity of nanomaterials. This concern comes from two major aspects. First, a number of nanomaterials have been applied in photodynamic therapies (Cheng et al., 2014), suggesting their toxicity under continuous irradiation. Second, under the continuous irradiation of STED light, some nanoprobes such as LPR hybrids showed a considerable photothermal effect (Cortés et al., 2016) that might damage the living samples, which should be taken into account in the toxicity assessment of STED nanoprobes. However, little work has been applied, except for individual reports on the low phototoxicity of PDots (Wu et al., 2020).

### Specific Targeting

There are two major strategies to create specific-targeting nanoparticles. The first one is the immunofluorescence method, which is a universal method that allows the specific targeting of interested subcellular structures such as microtubules. Small-sized (<30 nm) particles like AIE dots, PDots, ligand-modified QDots, and UCNPs have all been demonstrated for subcellular targeting by the immunofluorescence method (Fang et al., 2017; Wu et al., 2020; Hanne et al., 2015; Zhan et al., 2017; Urban et al., 2018) (see Table 4). However, the immunofluorescence method in most cases is adapted for fixed cell, as the transportation of antibodies across the membrane requires enhanced permeability. Another problem with immunofluorescence staining is that the steric effect of nanoparticles might induce discontinuous label, which might lead to a false-dotted signal, especially when higher imaging resolutions are achieved (Wu et al., 2020). It is also worth noting that the ability of immunofluorescence labeling is also limited by the size and dispersibility of materials, as silica particles and FNDs with aggregation tendency under physiological environments have proved not to be suitable for specific targeting so far (Schubbe et al., 2010; Schübbe et al., 2012; Prabhakar et al., 2013; Prabhakar et al., 2018).

**TABLE 4:** Summary of representative works on STED nanoprobes for bioimaging.

Categories	STED power	Resolution	Bioimaging applications	References
Dye-doped SiNPs	\	88 ± 4 nm	Cellular intake quantification	Peuschel et al. (2015)
	18∼38 mW (6.27 ∼13.23 MW/cm^2^)	19.2 nm (particles, 38 mW)	Nonspecific cell imaging	Liang et al. (2020)
		43.6 (*in vitro*, 18 mW)		
	0.89 MW/cm^2^	61∼65 nm (particles and *in vitro*)	Nonspecific cell imaging	Man et al. (2019)
AIE dots	100 MW/cm^2^	95 nm	Specific labeling (microtubule)	Fang et al. (2017)
	150 mW	74.37 nm	Specific labeling (mitochondria)	Li et al. (2018)
	144 mW	∼100 nm	Nonspecific cell imaging	Xu et al. (2020)
PDots	3 mW or 10 MW/cm^2^	78 nm	Specific labeling, real-time tracking, dual-color STED	Wu et al. (2020)
	3 mW or 10 MW/cm^2^	68 nm		
FNDs	180 mW	39 nm	Nonspecific cell imaging	Tzeng et al. (2011)
	130 MW/cm^2^	90 nm	Nonspecific cell imaging	Laporte and Psaltis, (2016)
QDots	150 mW	54 nm	Specific labeling (vimentin fiber)	Hanne et al. (2015)
	200 mW	85 nm	Specific labeling (microtubule)	Yang et al. (2016b)
	39.6 mW	21 nm	Nonspecific cell imaging	Ye et al. (2020)
	27.5 mW	20.6 nm	Nonspecific plant cell imaging	Ye et al. (2018)
CDots	\	71 (±25) nm	Lysosome imaging	Leménager et al. (2014)
	\	∼130 nm	Bacteria imaging	Yang et al. (2016a)
	39.6 mW	22.1 nm	Nucleus/tunneling nanotubes imaging	Li et al. (2019)
UCNPs	\	66 nm (particles)/82 nm (cellular skeleton)	Specific labeling (cellular skeleton)	Zhan et al. (2017)
LPR hybrids	0.5∼1.5 MW/cm^2^	20∼50% improved from confocal results*	Specific labeling (actin)	Cortés et al., (2016)

*The specific imaging resolution was not provided.

As the other option, active targeting in living cells is mostly facilitated by introducing charge and hydrophobicity/hydrophilicity through material design. For example, introducing hydrophobic cation structures might endow CDots and PDots with mitochondrial targeting ability (Li et al., 2018; Geng et al., 2019), while strong positive charge on an overall hydrophilic material might introduce affinity toward nucleic acids (Han et al., 2019). In some cases, the targeting of lysosomes or endosomes can also be achieved by utilizing the retention tendency of nanomaterials in these organelles (Leménager et al., 2014; Wu et al., 2020).

### Real-Time Tracking

Bioconjugated PDots have been applied to label endosomes with different caveolins and study their interaction in real time (Wu et al., 2020). Meanwhile, CDots and AIE dots with engineered surface charge have been used to perform real-time tracking on nucleus (Han et al., 2019) and mitochondrial (Li et al., 2018) activities (see Table 4).

The real-time tracking ability of STED nanoprobes is highly influenced by their imaging speed. Generally, a dwell time of <10 μs per pixel or several seconds per frame is considered acceptable for real-time tracking applications (Peng et al., 2019). For nanoparticles, two issues might decrease imaging speed and reduce their real-time tracking abilities. Firstly, STED light alone could excite the probes in some cases, which severely slows down the imaging acquisition as a second-time scanning, and thus, calculation is required to remove the background noise (Hanne et al., 2015). Fortunately, such inconvenience can be avoided by either selecting suitable STED wavelengths (Ye et al., 2020) or applying time-resolved methods for background elimination (Castello et al., 2017; Wang et al., 2018; Ma and Ha, 2019). The second issue that interferes with the STED imaging efficiency is the prolonged dwell time due to longer PL lifetime of materials. This is particularly significant for UCNPs, whose PL lifetime scales up to μs and leads to ultralong dwell time per pixel exceeding 1 ms (Liu et al., 2017). For the time being, this issue can only be addressed by accelerating emission kinetics through adjusting the material composition (Peng et al., 2019).

### Resolution and Power Requirement

The resolution of STED nanoprobes is limited by three major factors, namely, the size of particles, the depletion efficiency, and endurable imaging power. As discussed above, most reported STED nanoprobes have reached the size below 30 nm, providing little limit in the resolution of imaging (typically 30∼200 nm). The only exception was FNDs, which have achieved a higher resolution than particle size limitations (Rittweger et al., 2009; Wildanger et al., 2012). Still, considering the endurance of imaging samples toward STED irradiation, the bioimaging resolution was limited to ∼40 nm with a 180 mW STED power (Tzeng et al., 2011).

The power requirement and single-particle resolution of nanoprobes are basically determined by their saturation intensities. Materials like QDots, CDots, SiNPs, and UCNPs have achieved saturation intensities below 0.25 MW/cm^2^, allowing ∼30 nm single particle resolution with a low STED power of <50 mW or intensity <20 MW/cm^2^. As for AIE dots and LPR hybrid NPs, the power requirement for STED is typically 2∼4 times higher (100∼200 mW), while the overall resolutions are limited (∼70 nm), except for individual cases with ultrahigh STED power (312.5 mW, ∼30 nm) (Li et al., 2017). In comparison, the currently reported PDots for bioimaging typically require a very low STED power of <5 mW, while providing a final resolution of ∼70 nm (Wu Y. et al., 2018; Wu et al., 2020) (see Table 4).

### Multicolor STED Imaging

Multicolor STED imaging is a powerful tool for the study of nanoscale interactions in living organisms (Meyer et al., 2008). The successful multicolor STED imaging depends on two major conditions. First, the emission spectra of different probes should be distinguishable with minimal crosstalk. Secondly, identical STED and excitation wavelength should be applied for multicolor imaging if possible, in order to avoid locating error. For the first requirement, the emitting bandwidth of probes should be as narrow as possible (ideally <20 nm), which might be resolved by materials like QDs (Ye et al., 2018), UCNPs (Zhan et al., 2017), and some LPR hybrids (spaser) (Gao et al., 2020). The second requirement asks for large Stokes shifts, which can effectively avoid re-excitation and allow the application of a single STED wavelength for probes with different emissions. So far, this requirement has only been fulfilled by PDots (Wu et al., 2020), which however typically has a broad emission band.

## Conclusions and Outlook

Developing nanoprobes for STED imaging provides a valuable view on improving the STED imaging quality from a material perspective. These materials showed overall high brightness, photostability, and depletion efficiency. Furthermore, a variety of nanoprobes have demonstrated their applicability in realistic bioimaging of subdiffraction biostructures, both in fixed and living cells. Despite the abovementioned success, the steric effect, potential toxicity, and difficulties in modification of these materials still propose concern in their future development.

In order to reach the full potential of nanoprobe-based STED microscopy, the most important issue to address is perhaps their particle sizes. A reasonable future target for STED nanoprobes would be synthesizing particles with 2∼5 nm lateral size. The importance of the size might be important for STED imaging and could be explained as follows: first, considering the current single particle resolution limit of ∼20 nm, sub-5-nm particles in principle could have avoided significant discontinuous labeling in bioimaging. Second, it has been proved that sub-5-nm particles could be efficiently cleared from organisms after imaging (Soo Choi et al., 2007), which makes them safer imaging probes regardless of chemical composition. Third, smaller particles have a relatively larger surface area, which provides sufficient modifying spots for the tuning of their targeting ability. Meanwhile, it is also true that due to the size effect, particles smaller than 2 nm might become highly active and are hard to fabricate, stabilize, or modify. Also, the emission wavelength of particles like QDots and CDots is highly dependent on their size, and particles below 2 nm in diameter might show highly blue-shifted spectra, which would cause a higher phototoxicity in bioimaging.

Currently speaking, the development of STED nanoprobes is still in a primitive stage, where different types of nanomaterials are manufactured, tested, and measured in STED microscopy. However, introducing nanoprobes into STED should go beyond supplementing the library of fluorescent probe. Taking the unique chemical/physical properties of nanomaterials into account, we believe the vast potential of these materials in STED microscopy is yet to be fully realized.

### Exploring Imaging Applications for Microorganisms

Considering the growing concern on human health crisis caused by microorganisms, such as the occurrence of superbacteria with antibiotic resistance and the recent outbreak of coronavirus, real-time and superresolution bioimaging of microorganisms is becoming more and more important. STED imaging might provide crucial tools for systematic study of their behavior, infecting mechanism and potential cure, and the application of nanoprobes in this scenario is worth expecting. Currently, only CDots have been applied for bacteria STED imaging (Yang J. et al., 2016; Liu S. et al., 2020), and the potential of STED nanoprobes for microorganism imaging still needs further exploration.

### Utilizing Nanoprobes as Multifunctional Theranostic Platform

Compared with small molecules, nanoparticles are considered as more of platforms than fluorescent tags in their nature. Their microscopic size, large surface area, and improved stability are all in favor of creating multifunctional hybrid materials for theranostics applications (Kelkar and Reineke, 2011). It is promising to cooperate the subdiffraction STED imaging ability with mass delivery, catalysis, energy conversion, and photochemical functions which could allow *in situ* and real-time observation of the physical/chemical process, especially during a classical theranostic process with nanomaterials. The knowledge of biophysics at nanoscale acquired with this tool might deepen our understanding on the actual performance of nanomedicine and how to achieve better therapeutic effects.

### Nanoprobes for Multimode Superresolution Imaging

Subdiffraction imaging methods including STED, PALM, STORM, and SIM together with electronic microscopy provide an individual tool to achieve nanoscale resolution. However, cooperating different methods might provide further structural information of the interested biotargets. Many STED nanoprobes can also be utilized in other superresolution imaging methods (Dertinger et al., 2009; Yang X. et al., 2016; Chen et al., 2017; Zhi et al., 2018), which in principle allow multimode superresolution imaging with the same material (Denkova et al., 2019). Furthermore, inorganic nanomaterials like FNDs provide high contrast in TEM, which have enabled the cooperation of STED and TEM for cellular research (Prabhakar et al., 2018).

### From STED to Beyond

The STED imaging method itself undergoes fast development and has become very mature nowadays, as the resolution, imaging speed, and photon efficiency have greatly improved (Heine et al., 2017; Eilers et al., 2018). Meanwhile, novel imaging methods specifically applicable for nanoprobes relying on STED or STED-like imaging setups have been developed, most of which can be assigned to the concept of reversible saturable optical fluorescence transition (RESOLFT) imaging proposed by Hell et al. (Hofmann et al., 2005). By triggering fluorescence depletion through other saturation pathways (e.g., by exciting the fluorophore into a metastable dark state), the shrinking of PSF can be realized, in many cases, with much lower saturation intensities (1∼2 magnitude lower than the corresponding STED *I*_*sat*_). For many nanoprobes like QDots (Irvine et al., 2008; Xu et al., 2014; Kianinia et al., 2018), FNDs (Han et al., 2010), UCNPs (Kolesov et al., 2011; Wu et al., 2015), and CDots (Khan et al., 2015), such pathways have been discovered, and some were even utilized for STED-like subdiffraction imaging.

Another idea to perform STED-like imaging was to get rid of the central excitation beam and using the donut-shaped beam as the only excitation/saturation source. Resultantly, dark spot in a circle of dim light occurs, indicating the location of nanoparticles (Chen et al., 2018). Furthermore, the idea of STED-like microscopy can be broadened beyond fluorescence imaging, such as the suppression of scattering imaging (SUSI) method with plasmonic gold nanoparticles (Xu et al., 2018).

With all the abovementioned success, we firmly believe that the mutual development of nanomaterials and STED/STED-like imaging technique shall continuously provide new perspectives in achieving superresolved imaging both in space and time, shedding new lights into the subdiffraction bioimaging.
